# Obesity, Physical Activity, and Cancer Incidence in Two Geographically Distinct Populations; The Gulf Cooperation Council Countries and the United Kingdom—A Systematic Review and Meta-Analysis

**DOI:** 10.3390/cancers16244205

**Published:** 2024-12-17

**Authors:** Christine Gaskell, Stuart Lutimba, Ghizlane Bendriss, Eiman Aleem

**Affiliations:** 1 Premedical Division, Weill Cornell Medicine, Doha P.O. Box 24144, Qatar; gaskelc2@lsbu.ac.uk (C.G.); ghb2002@qatar-med.cornell.edu (G.B.); 2 Cancer Biology and Therapy Research Group, School of Applied Sciences, Division of Human Sciences, London South Bank University, 103 Borough Road, London SE1 0AA, UK; stuart.lutimba@lsbu.ac.uk

**Keywords:** cancer, obesity, physical activity, Gulf Cooperation Council Countries, United Kingdom, meta-analysis, systematic review

## Abstract

Background: The relationship between obesity, physical activity, and cancer has not been well studied across different countries. The age-standardized rate of cancer in the UK is double–triple that in the Gulf Cooperation Council Countries (GCCCs). Here, we study the association between obesity, physical activity, and cancer incidence with the aim to elucidate cancer epidemiology and risk factors in two geographically, ethnically, and climatically different parts of the world. Methods: Our systematic search (from 2016 to 2023) in PubMed, EMBASE, Scopus, and APA PsycINFO databases resulted in 64 studies totaling 13,609,578 participants. The Cochrane risk of bias tool, GRADE, R programming language, and the meta package were used. Results: Significant associations between obesity and cancer were found in both regions, with a stronger association in the UK (*p* ≤ 0.0001) than the GCCCs (*p* = 0.0042). While physical inactivity alone did not show a statistically significant association with cancer incidence, the pooled hazard ratio analysis revealed that the presence of both obesity and physical inactivity was associated with a significantly higher cancer incidence. The most common types of cancer were breast cancer in the UK and colorectal cancer across the GCCCs. Conclusion: Although both regions share similarities, advanced healthcare systems, genetic characteristics, dietary habits, and cultural practices may influence cancer incidence and types.

## Simple Summary

Approximately 40% of cancer is due to modifiable lifestyle factors including obesity and physical activity. Lifestyle factors vary in different populations, which may affect cancer incidence. The age-standardized rate of cancer in the UK is double–triple that in the Gulf Cooperation Council Countries (GCCCs). This study aimed to examine the association between obesity, physical activity, and cancer in both regions to elucidate cancer epidemiology and risk factors in two geographically and ethnically different regions. Significant associations between obesity and cancer were found in both regions. While there was no significant association between physical inactivity and cancer, the presence of both obesity and physical inactivity was associated with a significantly higher cancer incidence. Breast cancer was most common in the UK, while colorectal cancer was most common across the GCCCs. Our study identified gaps in the research from the GCCCs, highlighting the need for region-specific data to guide evidence-based policies aimed at preventing obesity, and increasing physical activity, to reduce cancer incidence.

## 1. Introduction

Cancer is the second-leading cause of death worldwide. Approximately 40% of cancer may be preventable by modifying lifestyle factors [1]. Lifestyle factors that affect the incidence and mortality of cancer include tobacco, alcohol consumption, diet, physical activity, exposure to infectious agents, environmental pollutants, and radiation [2]. Among the many contributing factors, the twin epidemics of obesity and physical inactivity have emerged as significant determinants of cancer risk [3,4]. Obesity often leads to chronic, low-level inflammation in the body [4,5], which can create an environment conducive to the development and growth of cancer cells. Obesity is linked to a number of different cancers including breast, colorectal, esophageal, kidney, gallbladder, uterine, pancreatic, and liver [4,6,7]. Inflammatory molecules can damage DNA and promote the formation of tumors [6,8,9,10,11,12]. Adipocytes, particularly in abdominal obesity, can produce hormones and growth factors, such as insulin and insulin-like growth factor-1 (IGF-1), in excess [13]. These hormones can stimulate the growth of cancer cells [2,14]. In postmenopausal women, obesity is linked to higher levels of estrogen, which is associated with an increased risk of breast and endometrial cancers [7,15]. Physical inactivity contributes to an increased risk of cancer, specifically endometrial, colon, and lung [16], through multiple mechanisms, such as promoting obesity [17], potentially altering sex hormone levels [18], increasing inflammation [19], and affecting immune function [20]. Regular physical activity has been associated with a decreased risk of various cancers, including breast, colon, and endometrial cancers [18,21,22]. It can also improve cancer-related outcomes in terms of survival and quality of life [3,23].

Modifiable lifestyle factors, such as obesity and physical activity, vary in different geographic regions of the world, which may impact cancer incidence. While ample research has been conducted in Western populations, the dynamics in the Middle East remain underexplored [24,25]. The Middle East countries with the lowest age-standardized rates (ASRs) of cancer are the Gulf Cooperation Council Countries (GCCCs) [26]. In contrast, the ASR/100,000 in the UK (296.1) is double to triple that in the six GCCCs (105.8 ± 7) [26]. Therefore, these two regions provide intriguing case studies for investigating the intricate relationship between these lifestyle factors and cancer. There is limited research that directly compares lifestyle and cancer incidence between the GCCCs and the UK. An innovative focus on these distinct regions will help to address gaps in global cancer epidemiology, offering geographically and culturally relevant insights, particularly in the under-researched GCCCs.

The GCCCs are comprised of Bahrain, Kuwait, Oman, Qatar, Saudi Arabia, and the United Arab Emirates (UAE) and have undergone a profound societal transformation over the past few decades [27]. This transformation has ushered in lifestyle changes, including shifts towards Western diets [24,28], which have had implications for health. The United Kingdom, on the other hand, has grappled with its own set of health challenges, including the prevalence of obesity with rates rising from 15% of the population in 1993 to 28% of the population in 2019 [29,30]. In our recent survey on the association of lifestyle factors with co-morbidities in Qatar and the UK, we found that the percentage of respondents in Qatar who eat out or order in meals 4+ times/week is 12.5% versus 3.6% in the UK [24]. Restaurant meals tend to be less nutritious and more processed than home-made meals, which may contribute towards obesity in Qatar [31,32,33]. The UK and GCCCs have similarities such as being high-income economies, culturally diverse with populations of varying ethnic cultures and religious backgrounds. Despite having diverse cultural populations, the cultural practices and norms are very different due to religious and social structures, and these differences may impact dietary habits and attitudes towards healthcare and treatment. The UK and the GCCC region both have modern healthcare and access to skilled healthcare professionals [34,35]. There are also many differences between the UK and GCCCs; firstly, the geography and climate, the UK has a temperate climate and mild temperatures. In contrast, the GCCCs have a desert climate, high levels of sun exposure, and extremely hot temperatures, making it very challenging for the residents to partake in sports/outdoor activities [24].

The goal of this study was to identify the region-specific association between obesity, physical activity, and cancer incidence with the aim of gaining insight into potential cancer epidemiology and risk factors in the two regions. Results from this study may help to identify lifestyle factors associated with the higher age-standardized rate of cancer in the UK compared to that in the GCCCs.

## 2. Materials and Methods

This systematic review followed the recommendations of the Preferred Reporting Items for Systematic reviews and Meta-Analyses (PRISMA) guidelines [36] (Supplementary Table S1a,b). The protocol has not been registered. The study also incorporates the Population, Intervention, Comparator, and Outcomes (PICO) framework (Supplementary Table S2).

### 2.1. Search Strategy

**Figure 1:**
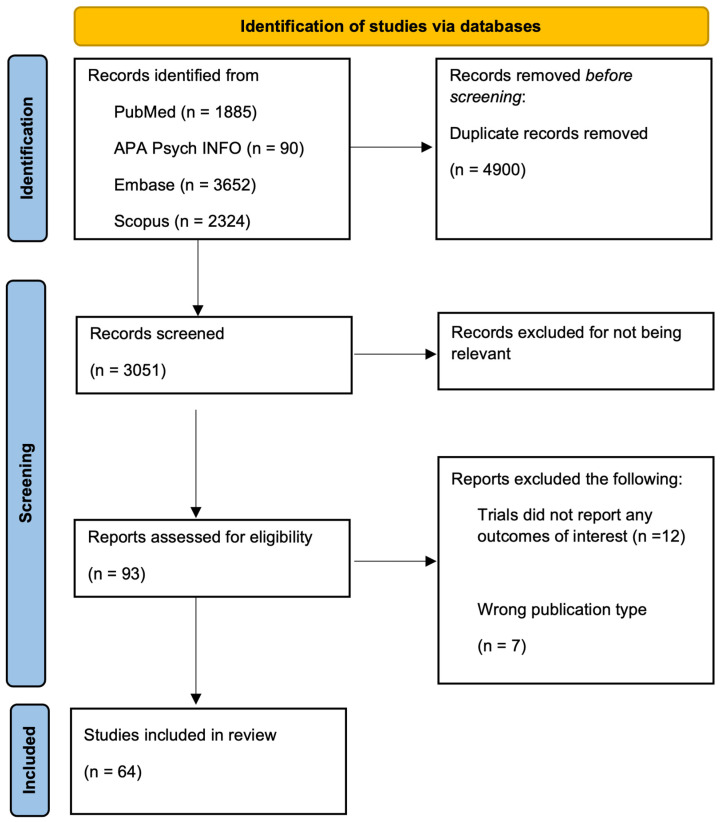
Flow chart of study selection for inclusion in the meta-analysis (PRISMA flow chart).

Literature searches were conducted in the following bibliographic databases on 13 November 2023: APA PsycINFO (EBSCOhost), Embase, PubMed (United States National Library of Medicine), and Scopus (Elsevier) with a total of 7951 articles collected. The overall structure for all searches was GCC/UK AND Cancer AND Obesity/Physical Activity. A range of synonyms were used for each concept in all searches, while the Embase and PubMed included appropriate Emtree and MeSH subject headings, respectively, without utilizing any restrictions like date, language, or study type. The complete details of all searches are shown in Supplementary Table S3 (Databases Search Strategies). Search results were downloaded as .NBIB (PubMed) or .RIS (APA PsycINFO, Embase, Scopus) files and uploaded to the Rayyan systematic review tool accessed 14 February 2024 (https://rayyan.ai/) for deduplication and screening (Figure 1).

### 2.2. Inclusion and Exclusion Criteria

Abstracts of all citations collected were reviewed and stored in the Rayyan software for systematic reviews (https://rayyan.ai/, accessed on 14 February 2024). The following inclusion criteria were used for study selection: (1) Cohort studies examining exposure to excess body weight or body mass index and the incidence or mortality of a cancer. (2) Studies containing an explicit description of BMI or obesity or overweight or physical activity or physical inactivity, with raw data on each group. (3) Inclusion of the country of study as being either the UK or one of the GCCCs. (4) The date of paper publication to be 2016 or after to ensure the most recent evidence was analyzed. We defined body mass categories using the following equation and ranges [BMI = weight (kg)/height (m^2^)]: normal (BMI = 18.5–<25), overweight (BMI = 25–<30), obese (BMI ≥ 30). For some studies, it was necessary to create a group that included both overweight and obese (BMI ≥ 25).

The exclusion criteria were (1) no raw data available; (2) duplicates; (3) no usable data reported; and (4) countries outside the UK and GCCCs.

### 2.3. Data Extraction

The data extraction and screening process followed a rigorous approach to ensure the accuracy and completeness of the information obtained from the studies included. Data were extracted independently by two reviewers to minimize biases and errors. The data extraction process involved capturing critical information from eligible studies including study characteristics (example, authors’ names, and publication year), participant characteristics (number of participants, age, country of residence, and gender), exposure variables (e.g., obesity and physical exercise), outcome measures (e.g., cancer incidence and cancer mortality), and effect estimates (e.g., hazard ratio). This comprehensive set of variables allowed for a thorough examination of the relationship between the exposure (e.g., obesity and physical activity) and the outcome (e.g., cancer incidence and survival) across various contexts and populations.

The data obtained (Table 1) served as a standardized repository for the extracted information. Upon loading the data into the statistical software environment R, necessary data transformations were performed to ensure the integrity and compatibility of the dataset for subsequent analyses [37]. Specifically, the column containing the sample size data was converted from its initial format (e.g., character and string) into numeric format.

#### 2.3.1. Hazard Ratio

We utilized the hazard ratio as the principal effect measure in the synthesis and presentation of results. This aligns with established practices for time-to-event data in cancer epidemiology studies, particularly in the context of cohort studies investigating the association between exposures, such as obesity and physical activity, and the incidence or mortality of various cancer types.

For studies that did not directly report hazard ratios, the hazard ratio values were derived or transformed from other relevant numerical parameters available in the article, such as risk ratios, odds ratios, or sufficient data to calculate hazard ratios indirectly. This step was crucial for conducting meta-analysis, which requires numerical input for variables like sample size and effect size measures.

The conversion of effect size estimators to a common hazard ratio was approached through several methods. For rare outcomes, hazard ratios (HRs) approximated risk ratios (RRs). In cases of common outcomes, the formula RR ≈ HR (1/[1 − P0(t)]) was used, where P0(t) represented the event rate in the unexposed group at time t. A more conservative estimate was obtained using the square root transformation: RR ≈ √HR. When converting from odds ratios (ORs) to RRs, the formula RR ≈ OR/(1 − P0 + (P0 × OR)) was applied, with P0 being the incidence of the outcome in the unexposed group. These conversions were interpreted cautiously, considering the specific study design and outcome prevalence [38,39].

In presenting the results of our analysis, we reported the pooled hazard ratios and their corresponding 95% confidence intervals for the associations between BMI categories (e.g., overweight and obese) or physical activity levels and the incidence of specific cancer types or overall cancer incidence. These effect estimates were obtained through meta-analytic techniques, such as random-effects or fixed-effects models, depending on the heterogeneity observed across the included studies (see below).

#### 2.3.2. Heterogeneity

In this meta-analysis, both fixed-effects and random-effects models were employed to synthesize data across studies. The fixed-effects model was used when the assumption was that all studies estimate the same underlying effect size, suitable for cases with minimal heterogeneity. Conversely, the random-effects model was applied when there was substantial heterogeneity among study results, indicating that the effect sizes vary across studies due to differences in study populations, interventions, or other factors. This model assumes that the observed effects are a random sample from a distribution of possible effects. Statistical heterogeneity was assessed using the *I*^2^ statistic, and appropriate models were chosen based on the degree of heterogeneity observed. Adjustments for potential confounders were made to ensure robust and reliable estimates of the overall effect size.

### 2.4. Risk of Bias

We assessed the risk of bias for every study included as defined in the PRISMA guidelines with the help of the Cochrane Collaboration risk of bias tool. Two reviewers assessed each study independently. The assessments included the following six domains: selection bias, performance bias, detection bias, attrition bias, reporting bias, and other bias. Each of the domains was judged as having low, high, or uncertain/unclear risk of bias. Results are summarized in Supplementary Figure S1a,b.

The GRADE tool was also utilized to assess the risk of bias in included studies (Supplementary Table S4).

#### 2.4.1. Overall Risk of Bias

The overall risk of bias for the studies included ranged from low to moderate. While most domains in most studies presented a low risk of bias, some were rated to raise some concern regarding selection bias and other biases due to geographical focus of this study.

#### 2.4.2. Strengths and Limitations

Major strengths of the studies included items like good reporting of procedures for blinding and handling of missing data. The weaknesses involved probable selection bias in some of the studies and intrinsic difficulties when comparing two geographically very distinct populations.

This overall risk of bias assessment provides context for interpreting the meta-analysis and systematic review. This means that although generally the quality of evidence is good, some care should be taken in drawing inference to any general conclusion, more specifically on the comparison between the two geographical regions.

### 2.5. Statistical Analysis and Synthesis Methods

Meta-analysis was conducted utilizing the R programming language and the meta package under the assumptions of both fixed-effects and random-effects models. The metagen function was employed, utilizing the inverse of the variance as weights. Additionally, custom weights derived from hazard ratios were computed and incorporated into the analysis. Subgroup analyses were performed based on variables such as body mass index (BMI) and ethnic diversity to explore potential disparities across different subpopulations. For each included study, we computed the effect size as the natural logarithm of the hazard ratio (log HR), which represents the measure of the effect of an exposure (e.g., obesity and physical activity) on an outcome (e.g., cancer incidence). The standard error was calculated as the reciprocal of the square root of the sample size. A *p*-value less than 0.05 was considered statistically significant. The results were expressed as hazard ratios (HRs) for the categorical variables, along with their corresponding 95% confidence intervals (CIs). The meta-analysis results were visually represented using forest plots, providing a concise depiction of the findings with diamonds denoting aggregate relative risks and squares representing study-specific relative hazards. The width of the diamonds represents the summary estimates’ 95% confidence intervals (CIs), while the area of each square is equivalent to the inverse of the variance of the logarithm of the relative risk for individual studies. Publication bias was evaluated through the utilization of funnel plots, with further statistical assessments conducted using methods such as metabias and trimfill to address any observed asymmetries or biases.

#### 2.5.1. Subgroup Analysis of the Study Population

The subgroup analysis aimed to investigate potential variations in the association between obesity, physical activity, and cancer risk in the UK and the GCCCs and age, gender in association with cancer incidence, and cancer type in association with obesity. Physical activity was evaluated utilizing Metabolic Equivalent of Task (MET) values, a standardized metric employed to quantify energy expenditure across various physical activities. The MET values were calculated based on the intensity, duration, and frequency of activities as reported in the selected studies and Supplementary Data. These quantified MET values, along with additional variables, were analyzed to investigate the association between physical activity and cancer incidence within the study populations. Statistical analyses were performed to control potential confounders and to identify statistically significant associations. By stratifying the meta-analysis based on country of residence, the goal was to identify disparities in effect sizes and elucidate subgroup-specific trends. Subgroup meta-analyses were performed using the meta package in R, with country of residence as a categorical variable for stratification. Statistical tests, such as Cochran’s Q test, Pearson correlation tests, and meta-regression, were employed to assess the significance of subgroup differences.

#### 2.5.2. Sensitivity Analysis

A multifaceted analytical strategy was implemented to comprehensively evaluate the obesity–cancer association across the GCCC and UK cohorts. This approach avoids reliance on *p*-values, which are sensitive to sample size. The hazard ratio with 95% confidence intervals (CIs) was our primary measure of effect, offering meaningful cross-population comparisons. Subgroup analyses were conducted to explore potential heterogeneity across demographic and clinical subpopulations. To minimize bias, ensure robustness of the quantitative synthesis of merged outcomes, and account for confounding factors, we adjusted for age, sex, and cancer type using multivariate Cox proportional hazards models. This robust methodology allows for a more nuanced and reliable assessment of the relationship between obesity and cancer, not bound by the limitations of *p*-values alone when comparing cohorts of different sizes.

## 3. Results

### 3.1. Study Selection

The initial literature search identified 7951 potentially relevant papers, 4900 duplicates were removed, and 3051 studies were subsequently screened through a systematic process of title, abstract, and full-text review. Two thousand nine hundred and fifty-eight studies were then excluded because they were irrelevant to this meta-analysis, ultimately yielding 64 cohort studies with a total population of 13,609,578 that met the inclusion criteria (Figure 1).

### 3.2. Study Characteristics

The included 64 studies had sample sizes varying substantially from 29 to 1,255,529 participants (Table 1). This heterogeneity in study scales reflects the broad scope of the research topic. The hazard ratios, a measure of relative risk, exhibited moderate variability across the studies, ranging from 0.05 to 1.89, with a mean of 0.7592 and an interquartile range of 0.56 to 0.91. This distribution suggests a range of effect sizes reported in the included studies.

The dataset incorporated studies from two geographically and culturally distinct regions, with the majority conducted in the United Kingdom (42 studies), followed by Saudi Arabia (13 studies), Oman (4 studies), Kuwait (2 studies), and one study each from Qatar, the United Arab Emirates, and one study from the Gulf Cooperation Council Countries (GCCCs) as one region. Regarding gender representation, the dataset exhibited a slightly higher mean number of female participants (approximately 117,472) compared to male participants (approximately 96,772), with minimum values of zero for both genders, suggesting the potential absence of gender-specific data in some observations.

The physical exercise levels, a significant factor in obesity studies, had a mean of 0.462 and an interquartile range of 0.25 to 0.66, indicating moderate variability across the studies. The range from 0.01 to 1.0 suggests substantial variability, with some studies reporting very low activity levels (0.01) and others near the maximum (1).

**Table 1:** Characteristics of studies included in this meta-analysis.

Author Year	Sample Size	HR	Origin of Study	PA Kcal/kg/h	Cancer Class	BMI	Age Group	Mean Age
Malcomson F. C. et al., 2024 [40]	288,802	0.93	UK	0.5	Breast, GI, Respiratory, Genitourinary	N/A	50s–70s	56.2
Yu Y. et al., 2023 [41]	170,726	0.92	UK	0.88	Genitourinary, GI, Respiratory, Endocrine, Breast, Head and Neck	15	30s–50s	48.5
Maroto-Rodriguez et al., 2024 [42]	110,799	0.83	UK	0.98	Nonspecific	N/A	Wide Range	57.6
Safizadeh et al., 2023 [43]	453,049	1.23	UK	0.17	GI	30	40s–60s	54.4
Sweetland et al., 2023 [44]	1,255,529	1.12	UK	0.85	GI	22.5	50s–70s	56.7
Rask-Andersen et al., 2023 [45]	442,519	0.97	UK	0.31	GI, Skin and Soft Tissue, Head and Neck, Breast, Neurological, Genitourinary, Endocrine, Respiratory	27.5	Wide Range	55
Al Shareef Z. et al., 2023 [46]	192	1.35	UAE	0.48	Genitourinary	25	50s–70s	61
Maina J. G. et al., 2023 [47]	457,270	1.08	UK	0.38	GI	25	Wide Range	58.4
Chen J. et al., 2023 [48]	408,815	1.07	UK	0.39	Genitourinary, GI, Breast, Head and Neck, Skin and Soft Tissue, Endocrine, Neurological	30	Unspecified	N/A
Hu W. et al., 2023 [49]	205,654	1.52	UK	0.24	Nonspecific	30	Wide Range	45.5
Alrobaiq B. M. et al., 2023 [50]	137	1.89	Saudi Arabia	0.52	Genitourinary	29.64	50s–70s	57
Babiker et al., 2020 [51]	305	0.54	Saudi Arabia	0.46	Breast	30	40s–60s	46.5
Al-Lawati N.A. et al., 2020 [52]	1285	0.78	Oman	0.66	Endocrine	N/A	Wide Range	41
Al-Zalabani A. 2020 [53]	124	0.81	Saudi Arabia	0.48	GI	N/A	Unspecified	N/A
Alsolami F. J. et al., 2019 [54]	432	0.46	Saudi Arabia	0.25	Breast	35.4	Wide Range	57
Sulieman I. et al., 2019 [55]	35	0.81	Qatar	0.35	GI	27.1	50s–70s	52.3
Alomair A. O. et al., 2018 [56]	29	0.55	Saudi Arabia	0.64	GI	27.5	Wide Range	57.3
He Q. et al., 2023 [57]	375,998	0.86	UK	0.68	Genitourinary	41	Wide Range	55
Liang et al., 2023 [58]	92,221	0.96	UK	0.66	Nonspecific	29.75	50s–70s	62.4
Wang et al., 2023 [59]	81,882	0.5	UK	0.36	Genitourinary	N/A	Wide Range	55.2
Cao Y. et al., 2023 [60]	245,009	0.82	UK	0.29	Breast	N/A	Wide Range	54.7
Cao Z. et al., 2023 [61]	401,189	0.86	UK	0.77	Genitourinary, GI, Respiratory, Skin and Soft Tissue, Neurological, Breast, Hematologic, Endocrine, Head and Neck	N/A	Wide Range	53.7
Ahmadi et al., 2022 [62]	71,893	0.87	UK	0.1	Nonspecific	27.1	50s–70s	62.5
Ke et al., 2022 [63]	502,413	0.64	UK	0.83	GI	30	50s–70s	56.5
Yu E.Y-W. et. al., 2022 [64]	484,520	0.52	UK	0.16	Genitourinary	N/A	50s–70s	55.5
Ding et al., 2022 [65]	346,627	0.44	UK	0.56	Nonspecific	N/A	40s–60s	50
Yang et. al., 2022 [66]	342,079	0.83	UK	0.13	Breast	28	Unspecified	N/A
Swerdlow et al., 2022 [67]	3595	0.53	UK	0.28	Breast	32	Wide Range	45
Amin et al., 2022 [68]	331,924	0.84	UK	0.02	Breast, Genitourinary	37	40s–60s	56
Parra-Soto et al., 2022 [69]	442,610	0.96	UK	0.59	Respiratory, GI, Breast	25	Wide Range	55
Xia et al., 2021 [70]	474,929	0.89	UK	0.34	GI	29.12	40s–60s	54
Ahmed, 2021 [71]	321,472	0.68	UK	0.35	Nonspecific	30	Wide Range	55
Vithayathil et al., 2021 [72]	367,561	0.75	UK	0.64	GI, Respiratory, Genitourinary, Hematologic, Endocrine, Neurological, Breast, Skin and Soft Tissue, Head and Neck	27.4	50s–70s	57.2
Aivaliotis, 2021 [73]	34,493	0.91	UK	0.32	Breast	30	Unspecified	N/A
Christakoudi et al., 2021 [74]	430,615	0.8	UK	0.57	GI, Genitourinary, Breast	27.05	40s–60s	55
Huang et al., 2022 [75]	380,055	0.94	UK	0.32	Respiratory	40	50s–70s	55.9
Parra-Soto et al., 2021 [76]	437,393	0.9	UK	1	GI, Genitourinary, Breast	27.87	50s–70s	56.3
Sanikini et al., 2020 [77]	458,713	0.78	UK	0.2	GI	49.5	Wide Range	55.7
Cao Z. et al., 2020 [78]	390,575	0.84	UK	0.7	Genitourinary, GI, Breast, Hematologic, Respiratory	N/A	Wide Range	55.2
Murray et al., 2020 [79]	364,899	0.58	UK	0.5	Respiratory, Breast, GI	N/A	Wide Range	56
Rasmy, 2020 [80]	80	0.28	Saudi Arabia	0.57	Breast	55	50s–70s	50
Guo et al., 2020 [81]	174,160	0.76	UK	0.36	Breast	27	40s–60s	50
Hillreiner et al., 2020 [82]	59,191	0.37	UK	0.53	GI	15.3	Unspecified	N/A
Gharahkhani et al., 2019 [83]	46,155	0.57	UK	0.12	Nonspecific	25	30s–50s	54
Alsheridah, 2018 [84]	309	0.35	Kuwait	0.19	GI	30	40s–60s	52
Mafiana et al., 2018 [85]	270	0.66	Oman	0.34	GI	24	Wide Range	48
Kunzmann et al., 2018 [86]	359,033	0.48	UK	0.53	GI	30	Wide Range	47.5
Morris et al., 2018 [87]	430,584	0.2	UK	0.64	GI	30	40s–60s	54
Ortega et al., 2017 [88]	472,526	0.78	UK	0.76	GI	30	40s–60s	54
Karim et al., 2016 [89]	45	0.05	Saudi Arabia	0.33	Breast	32.5	Wide Range	48
Inan-Eroglu et al., 2023 [90]	399,575	0.82	UK	0.86	Nonspecific	28.3	40s–60s	53.7
Swerdlow et al., 2021 [91]	3595	0.65	UK	0.14	Breast	27.5	40s–60s	45
Renehan et al., 2020 [92]	47,042	0.55	UK	0.12	Breast	23.4	Wide Range	60
Alkhaldy, 2020 [93]	30	0.41	Saudi Arabia	0.61	GI	26	50s–70s	57
Alazzeh, 2018 [94]	301	0.71	Saudi Arabia	0.8	GI	27.2	Wide Range	56
Otaibi, 2017 [95]	180	0.47	Saudi Arabia	0.8	Breast	29.4	50s–70s	41
Zacharakis, 2023 [96]	35,640	0.33	Saudi Arabia	0.35	GI	25	40s–60s	62.2
Ramadan, 2023 [97]	328,575	0.34	GCCCs	0.04	GI	N/A	Wide Range	47
Abdelnaby et al., 2021 [98]	1005	0.43	Kuwait	0.22	GI	N/A	30s–50s	54
Al badi, 2020 [99]	465	0.16	Oman	0.88	Breast	N/A	40s–60s	N/A
Alshamsan et al., 2020 [100]	2212	0.03	Saudi Arabia	0.01	Breast	30	Unspecified	45
Radwi, 2019 [101]	95	0.67	Saudi Arabia	0.73	GI	30	40s–60s	48.4
Al-Lawati, 2018 [102]	232	0.85	Oman	0.24	GI	N/A	50s–70s	54.5

BMI—body mass index; GCCCs—Gulf Cooperation Council Countries; GI—gastrointestinal; HR—hazard ratio; PA—physical activity; N/A refers to the papers that did not report BMI, only physical activity; UK: United Kingdom.

### 3.3. Quality Assurance

The quality assurance of the evidence provided in this meta-analysis was evaluated based on the Grading of Recommendations Assessment, Development, and Evaluation (GRADE) (Supplementary Table S4). The table demonstrates a range of evidence quality, with many high-quality studies showing strong evidence for associations between various factors (e.g., diet, physical activity, and obesity) and the incidence of cancer. However, it also includes studies with lower quality rating due to limitations in study design or execution.

### 3.4. Association Between Obesity and Overall Cancer Incidence in Both Regions

**Figure 2:**
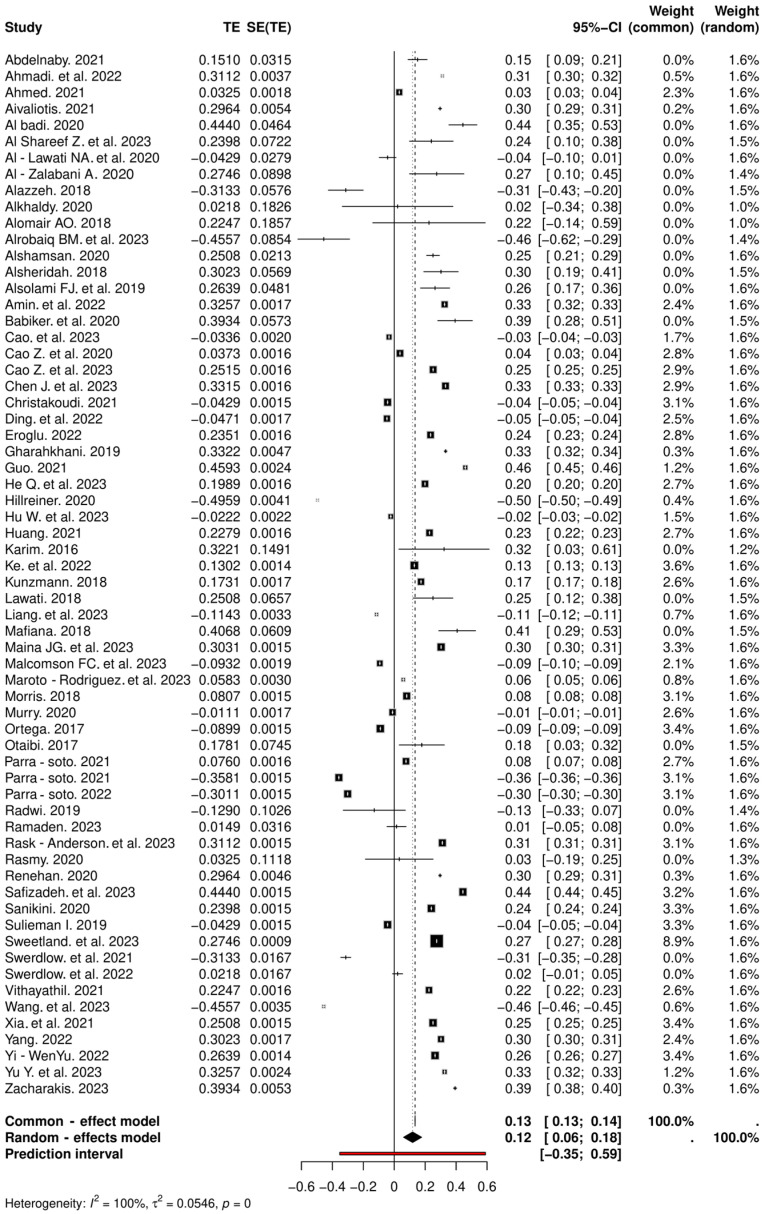
Forest plot of all studies showing the association of obesity and cancer incidence in both GCCCs and the UK. The random-effects model was used to adjust for heterogeneity. The black squares and lines represent the confidence intervals of the individual studies, the grey squares represent the study weight, and the grey diamond represents the pooled HR. CI, confidence interval, GCCCs, Gulf Cooperation Council Countries, HR, hazard ratio, SE, standard error, TE, treatment effect, UK, United Kingdom [40,41,42,43,44,45,46,47,48,49,50,51,52,53,54,55,56,57,58,59,60,61,62,63,64,65,66,67,68,69,70,71,72,73,74,75,76,77,78,79,80,81,82,83,84,85,86,87,88,89,90,91,92,93,94,95,96,97,98,99,100,101,102].

**Figure 3:**
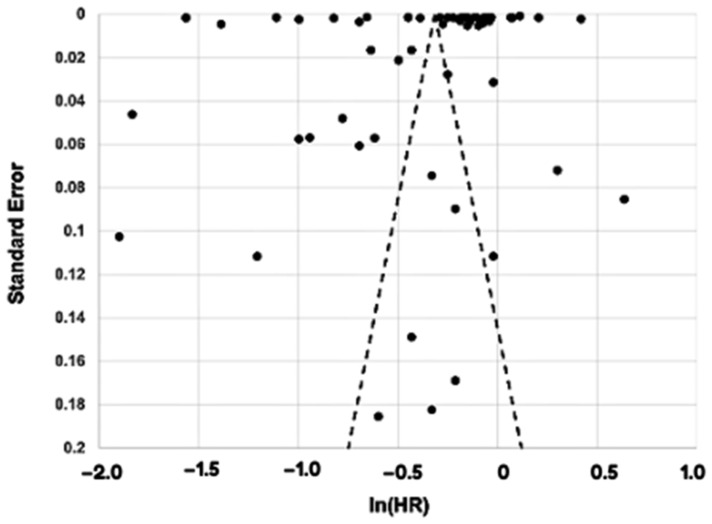
Funnel plot of all studies included in the meta-analysis. The *x*-axis displays the study estimated effect size with inverse hazard ratio (In(HR)), and the *y*-axis represents a measure of study precision, with standard error. The dots represent the effect sizes from individual studies plotted against their precision while the dashed lines signify the expected distribution of these studies. The distribution of the studies observed in the funnel plot could be due to the heterogeneity of the studies.

The meta-analysis included a total of 64 studies. The pooled hazard ratio from the random-effects model was 0.1188 (95% CI: 0.0605, 0.1771), indicating a statistically significant association between obesity and increased cancer risk. However, the analysis also revealed substantial heterogeneity, with an *I*^2^ statistic of 100% and a highly significant test of heterogeneity *(p* ≤ 0.0001) (Figure 2 and Figure 3). This suggests that the effect sizes varied considerably across the included studies, potentially due to differences in study populations, cancer types, or other factors. Positive associations (*p* ≤ 0.0001) were found between obesity and cancer in both the GCCC region and the United Kingdom as summarized in Table 1 and Figure 2.

Using the Trim-and-Fill method to account for potential publication bias, the adjusted hazard ratio remains significant, indicating a robust positive relationship between obesity and cancer risk. The random-effects model analysis yielded a pooled hazard ratio of 0.1188 [95% CI: 0.0605; 0.1771], demonstrating a statistically significant association between obesity and increased cancer risk (z = 3.99, *p* ≤ 0.0001). Considerable heterogeneity was observed among the studies (*I*^2^ = 100.0%, tau^2^ = 0.0546 [95% CI: 0.0388; 0.0801]), underscoring the variability in effect sizes across different populations. The small-study effects using the Q-Q′ test, revealed significant asymmetry (Q-Q′ = 1973.23, *df* = 1, *p* ≤ 0.0001). This suggests potential bias in smaller studies, which was further explored using the Trim-and-Fill method to adjust for these effects.

#### 3.4.1. Obesity and Cancer in the UK

**Figure 4:**
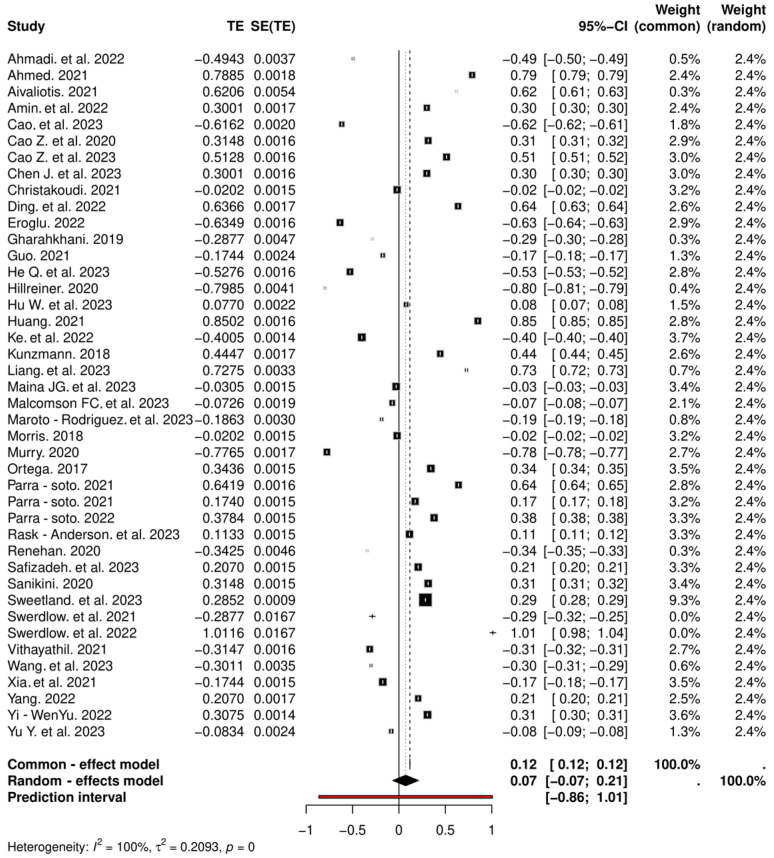
Forest plot of 42 studies showing the association of obesity and the incidence of cancer in the UK. The random-effects model was used to adjust for heterogeneity. The black squares and lines represent the confidence intervals of the individual studies, the grey squares represent the study weight, and the diamond represents the pooled HR. CI, confidence interval, HR, hazard ratio, SE, standard error, TE: treatment effect [40,41,42,43,44,45,47,48,49,57,58,59,60,61,62,63,64,65,66,67,68,69,70,71,72,73,74,75,76,77,78,79,81,82,83,86,87,88,90,91,92].

The meta-analysis included 42 studies from the UK region. The pooled hazard ratio from the random-effects model was 0.1057 (95% CI: 0.0337, 0.1777), indicating a statistically significant association between the exposure (obesity) and increased cancer risk in the UK. However, the analysis also revealed substantial heterogeneity, with an *I^2^* statistic of 100% and a highly significant test of heterogeneity (*p* ≤ 0.0001). This suggests that the effect sizes varied considerably across the UK studies included. The common-effects model, which assumes a uniform true effect size across all studies, resulted in a pooled hazard ratio of 0.1399 (95% CI: 0.1394, 0.1404), which is higher than the random-effects estimate. This discrepancy highlights the importance of considering the random-effects model, which accounts for the observed heterogeneity in the data (Figure 4).

The meta-analysis utilizing the inverse variance method revealed a statistically significant positive association between obesity and cancer risk within the UK subgroup, as evidenced by an overall effect size of 0.2295 (95% CI: 0.0746 to 0.3845, z = 2.90, *p* = 0.0037). Despite the robustness of this association, the Q-test for heterogeneity indicated substantial variability across studies (Q = 3721773.19, *df* = 49, *p* ≤ 0.0001). This high heterogeneity underscores the significant differences in effect sizes among the included studies. Additionally, the implementation of the Trim-and-Fill sensitivity method to address potential publication bias showed that all studies fell within the region of marginal statistical significance (*p* ≤ 0.10), suggesting that the observed association may be influenced by underlying biases in the included studies.

#### 3.4.2. Obesity and Cancer in the GCCC Region

**Figure 5:**
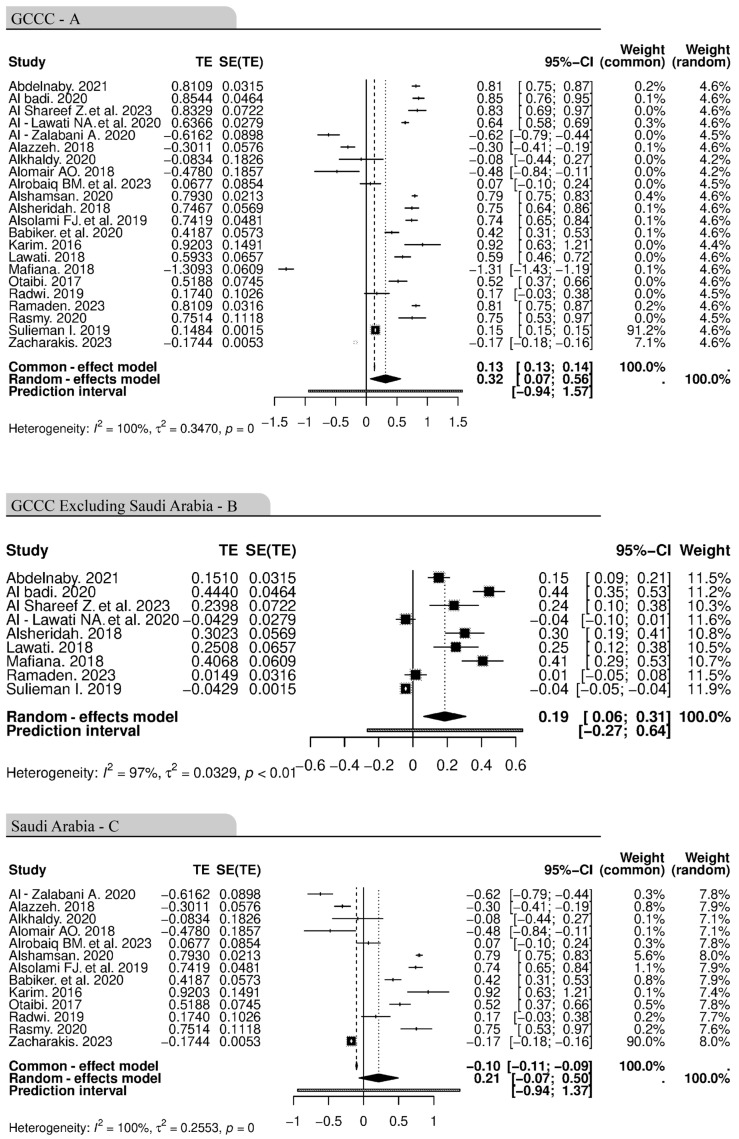
Forest plot showing the association of obesity and the incidence of cancer of 22 studies of the GCCCs (**A**), of 9 studies of the GCCCs excluding Saudi Arabia (**B**) and of 13 studies of Saudi Arabia (**C**). The random-effects model was used to adjust for heterogeneity. The black squares and lines represent the confidence intervals of the individual studies, the grey squares represent the study weight, and the grey diamond represents the pooled HR. CI, Confidence interval, GCCCs, Gulf cooperation countries council, HR, hazard ratio, SE, standard error, TE: treatment effect [46,50,51,52,53,54,55,56,80,84,85,89,93,94,95,96,97,98,99,100,101,102].

The meta-analysis included 22 studies from the GCCC region. Due to the large number of studies from Saudi Arabia in comparison to the rest of the GCCC region, we created three separate analyses for the GCCC region: GCCC region including Saudi Arabia (Figure 5A), GCCCs excluding Saudi Arabia (Figure 5B), and Saudi Arabia (Figure 5C). The summary of the analysis is found in Table 2.

The pooled hazard ratio from the random-effects model was 0.1467 (95% CI: 0.0462, 0.2472), indicating a statistically significant association between the exposure (obesity) and increased cancer risk in the GCCCs, including Saudi Arabia (Figure 5A). Statistically significant association between obesity and cancer incidence was also found in the nine studies from the GCCCs, excluding Saudi Arabia. The pooled hazard ratio from the random-effects model was 0.1857 (95% CI: 0.0632, 0.3083) (Figure 5B). The meta-analysis included 13 studies from Saudi Arabia (Figure 5C). The pooled hazard ratio from the random-effects model was 0.1137 (95% CI: −0.0390, 0.2663), indicating a statistically significant association between obesity and increased cancer risk in Saudi Arabia as well.

Substantial heterogeneity was found in studies from the three GCCC cohorts, with an *I*^2^ statistic of 99.7% for the GCCCs including Saudi Arabia, with an *I*^2^ statistic of 97.1% for the GCCCs excluding Saudi Arabia, and with an *I*^2^ statistic of 96% for the 13 studies from Saudi Arabia. Heterogeneity was highly significant in the three cohorts (*p* ≤ 0.0001), indicating that the effect sizes varied considerably across the included studies, potentially due to differences in study populations, cancer types, or other factors.

### 3.5. Association Between Age and Cancer Incidence

**Figure 6:**
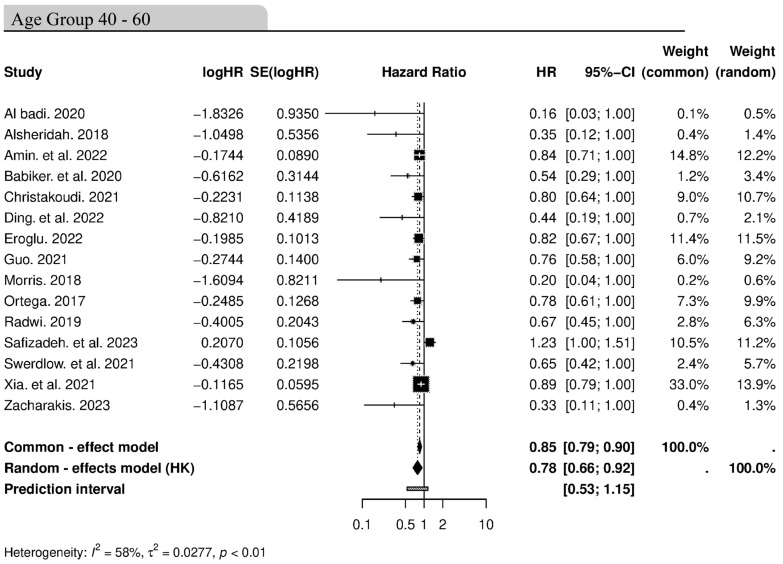
Forest plot illustrating the association between cancer incidence and age group (40–60). The black squares and lines represent the confidence intervals of the individual studies; the grey squares represent the study weight. The diamond at the bottom of the plot represents the overall pooled effect size, with its width reflecting the 95% CI. CI, confidence interval, HR, hazard ratio, SE, standard error [43,51,65,68,70,74,81,84,87,88,90,91,96,99,101].

We next assessed the association between cancer incidence and age groups. We divided the age groups reported in the different studies into the following: 30–50, 40–60, 50–70, unspecified, and wide range. The effect size from the random-effects model was 0.78 (95% CI: 0.66–0.92), indicating a statistically significant reduction in cancer risk for individuals in the 40–60 age range (Figure 6). This could suggest that being in the 40–60 age group is associated with a lower likelihood of adverse cancer outcomes. Heterogeneity with an *I*^2^ statistic of 58% (*p* ≤ 0.01) indicates that the effect sizes varied moderately across the included studies, potentially due to differences in study populations, cancer types, or other factors. The common-effects model supports the output from the random-effects model, yielding an effect size of 0.85 (95% CI: 0.79–0.90). The majority of the studies are found near the line of no effect, showcasing consistency across different studies (Figure 6). The association between cancer incidence and other age groups is presented in Supplementary Figure S2.

**Figure 7:**
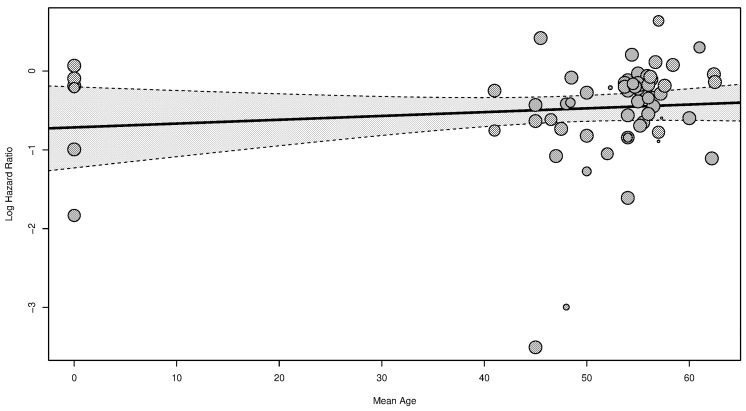
Meta-regression bubble plot showing the relationship between mean participant age and log hazard ratio (effect size). Each bubble represents a study, with bubble size proportional to the study’s weight in the meta-analysis. The solid line indicates the regression line, while the dashed lines represent the 95% confidence interval.

To address potential age-related heterogeneity, we conducted a meta-regression analysis using the mean age of participants in each study as a moderator. The results showed a slight but statistically significant moderation by age (*β* = −0.0082, *p* = 0.0487), suggesting that the mean age of participants had a small influence on the effect sizes observed across studies (Figure 7).

The test for residual heterogeneity was significant (*Q* = 283.64, *df* = 62, *p* ≤ 0.0001), indicating that there remains unexplained heterogeneity in our meta-analysis. The residual heterogeneity measure (***τ*^2^** = 0.1872) and the *I***^2^** statistic (78%) confirm that a moderate to high portion of variability is not accounted for by the mean age as a moderator. Additionally, the proportion of heterogeneity explained by the model (*R*^2^ = 12.35%) suggests that age contributed modestly to reducing the observed heterogeneity.

### 3.6. Association Between Gender and Cancer Incidence

**Figure 8:**
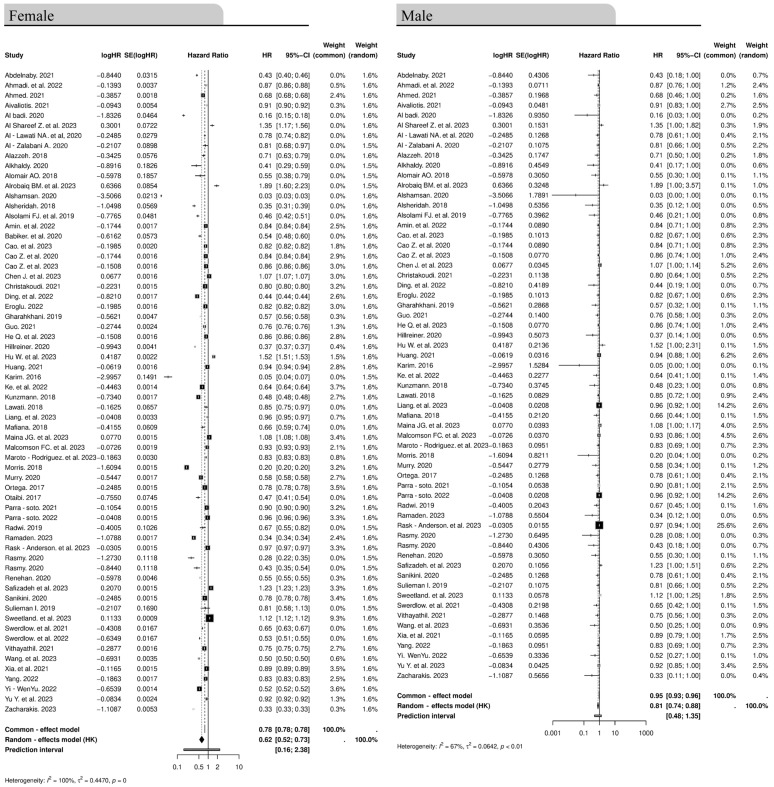
Forest plots show the association of gender and incidence of cancer for females and males [40,41,42,43,44,45,46,47,48,49,50,51,52,53,54,55,56,57,58,59,60,61,62,63,64,65,66,67,68,69,70,71,72,73,74,75,76,77,78,79,80,81,82,83,84,85,86,87,88,89,90,91,92,93,94,95,96,97,98,99,100,101,102].

We next assessed the association between gender and cancer incidence. The genders reported in all the studies used in the present meta-analysis were males and females (Figure 8). The random-effects model shows a moderate association with an effect size of 0.62 (95% CI: 0.52–0.73), while the common-effects model has a higher effect size of 0.78 (95% CI: 0.78–0.78). The high heterogeneity (*I*^2^ = 100%, *p* ≤ 0) among female studies indicates possible inconsistency. For males, the random-effects model demonstrates a somewhat stronger association with an effect size of 0.81 (95% CI: 0.74–0.88), and the common-effects model shows an even higher effect size of 0.95 (95% CI: 0.93–0.96). Male studies exhibit moderate to substantial heterogeneity (*I^2^* = 67%, *p* ≤ 0.01). However, despite the effect sizes shown, the forest plots demonstrate little effect of gender on the risk of cancer, with the studies close to the null effect line. In brief, no significant association between gender and cancer incidence was detected.

The studies are close to the line of null effect highlighting that there is almost no effect of gender on cancer incidence. The diamond at the bottom of the plot represents the overall pooled HR. CI, confidence interval, HR, hazard ratio, SE, standard error.

### 3.7. Association Between Cancer Type and Obesity

**Figure 9:**
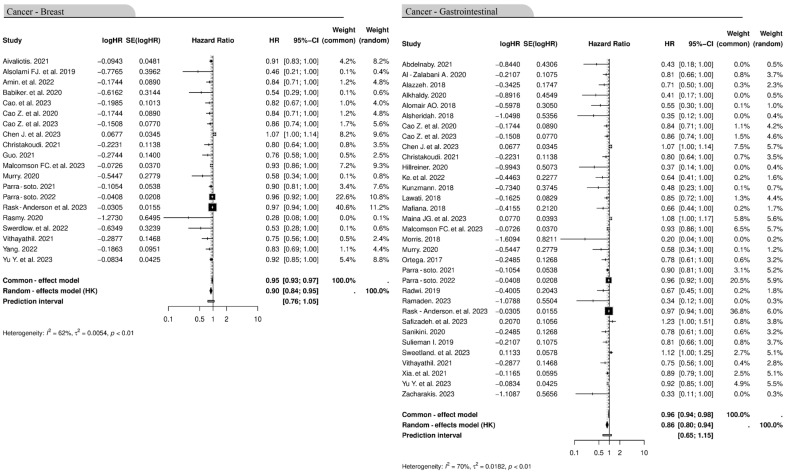
Forest plot showing the association of obesity and the incidence of breast and gastrointestinal cancer types. Both types of cancer had a statistically significant association with obesity. The diamond at the bottom of the plot represents the overall pooled HR. CI, confidence interval, HR, hazard ratio, SE, standard error [40,43,45,47,48,51,53,54,55,56,60,61,63,64,66,67,68,69,70,72,73,74,76,77,78,79,80,81,82,84,85,86,87,88,93,94,96,97,98,102].

We next assessed the association between cancer types and obesity. Breast cancer is the most common cancer in the UK and colorectal cancer in the GCCCs. For breast cancer, of the 20 studies included, the random-effects model yielded an effect size of 0.90 (95% CI: 0.84–0.95), indicating a statistically significant link between obesity and breast cancer incidence. Moderate heterogeneity was observed (*I*^2^ = 62%, *p* ≤ 0.01); this suggests that there was considerable variability among studies. The common-effects model showed similar findings with an effect size of 0.95 (95% CI: 0.93–0.97). The 33 studies that looked at gastrointestinal cancer highlighted more of an association with obesity. The random-effects model produced an effect size of 0.86 (95% CI: 0.80–0.94), while the common-effects model yielded 0.96 (95% CI: 0.94–0.98). Heterogeneity was slightly higher for gastrointestinal cancer studies (*I*^2^ = 70%, *p* ≤ 0.01). The results from the studies collected demonstrate a significant relationship between obesity and increased incidence for both cancer types, with gastrointestinal cancers showing a marginally stronger association (Figure 9). The association between obesity and cancer incidence of additional cancer types is shown in Supplementary Figure S3.

### 3.8. Association Between Physical Activity and Cancer Incidence

**Figure 10:**
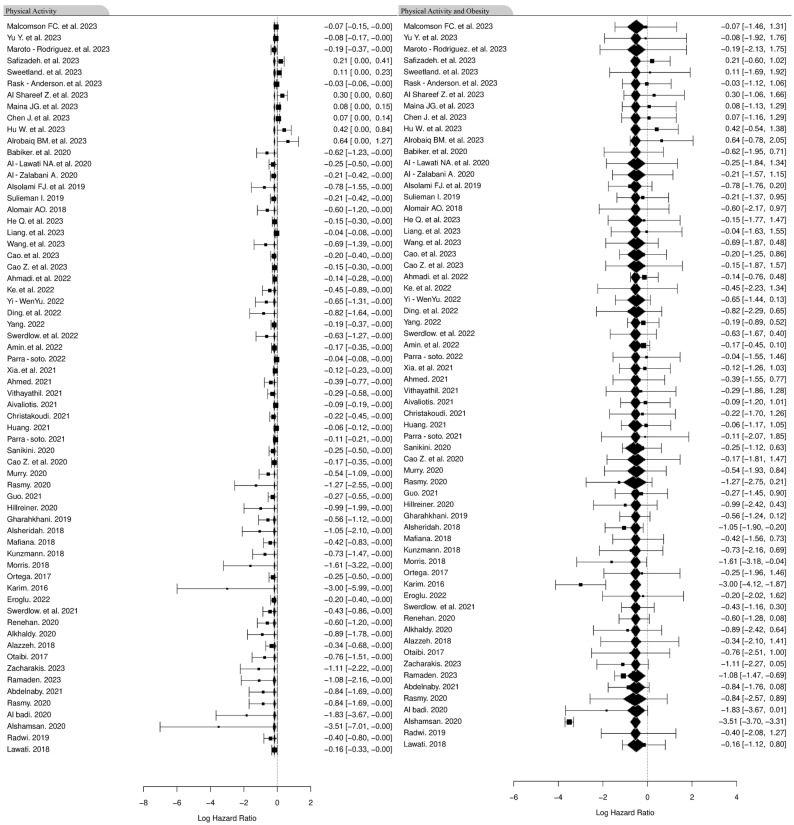
Forest plot showing the results of a mixed-effects meta-analysis model, synthesizing data from 64 studies using the Restricted Maximum Likelihood (REML) method to estimate variance components. The model fit statistics include a log likelihood of −22.5703, deviance of 45.1406, Akaike Information Criterion (AIC) of 51.1406, Bayesian Information Criterion (BIC) of 57.5220, and a Corrected AIC (AICc) of 51.5544. Heterogeneity measures indicate substantial variability among studies, with τ^2^ (residual heterogeneity) at 0.0194 (SE = 0.0064), *I*^2^ at 80.29%, and H^2^ at 5.07. A significant residual heterogeneity is evident from the Q_E statistic (Q_E(*df* = 62) = 193.2016, *p* ≤ 0.0001). However, the moderator effect of physical exercise is not significant (Q_M(*df* = 1) = 0.0266, *p* = 0.8705) [40,41,42,43,44,45,46,47,48,49,50,51,52,53,54,55,56,57,58,59,60,61,62,63,64,65,66,67,68,69,70,71,72,73,74,75,76,77,78,79,80,81,82,83,84,85,86,87,88,89,90,91,92,93,94,95,96,97,98,99,100,101,102].

We next assessed the association between physical activity and cancer (Figure 10). We found an insignificant association in the 95% confidence interval of 0.3 between those who engage in physical activity and the absence of cancer. When considering differences between genders (as reported in the studies), males showed a small association of 0.157 and females of 0.15 between physical inactivity and cancer. A Welch *T*-test showed that there is a significant difference in physical activity levels between males and females (*p* ≤ 0.01) with the sample estimates for males being 0.495 and 0.326 for females.

The presence of both obesity and lack of physical activity was shown to significantly increase the incidence of cancer (*p* ≤ 0.0001). The studies examined showed a statistically significant effect of physical activity on BMI (*p* ≤ 0.001). However, gender did not result in statistically significant difference (*p* = 0.45) when looking at the effect of both obesity and physical inactivity.

## 4. Discussion

The results of this meta-analysis demonstrate a significant association between obesity and cancer in both the UK and the GCCC region, with the magnitude of association being greater in the UK (*p* ≤ 0.0001) than the GCCC region (*p* = 0.0042). Although our study did not show a significant association between the lack of physical activity and cancer incidence, the presence of both factors suggests that individuals who are both obese and not physically active may face an increased incidence in their susceptibility to certain cancers, compared to those with either factor alone. This is consistent with previous findings indicating that higher levels of leisure-time physical activity are associated with lower incidence of 13 different types of cancer [103] and that a good quality diet combined with sufficient levels of physical activity are important for reducing the incidence of diet and adiposity-related cancer mortality [65].

The most common cancer associated with obesity from the studies analyzed for the UK was breast cancer and for the GCCC region was colorectal cancer. There have been multiple previous studies documenting the association between obesity and cancer in the UK population. For example, breast cancer in postmenopausal women [60], endometrial cancer, the most common gynecological cancer in high income countries [104], and esophageal cancer have an increased association when an individual has a BMI ≥ 30 in the UK [44]. It is noteworthy to mention that body fat distribution, which differs between sexes, is suggested to be an independent risk factor to cancer. For example, body fat distribution changes between post and premenopausal women changed the risk for a woman developing breast cancer [60]. Rask-Andersen et al. (2023) studied the effects of body fat accumulation and distribution on sex-specific cancer risk. They reported that body fat accumulation is associated with a larger number of cancers compared to fat distribution and that the effects of both differed between sexes in the case of colorectal, esophageal, and liver cancer [45]. In contrast, a study by Amin et al. (2022) suggested that increased adiposity has a protective effect against breast and prostate cancer [68].

Gastric cancer was also found to have increased association when an individual has a BMI ≥ 30 kg in comparison to BMI 18.5–25 [105]. A review conducted in the UK also highlighted a strong association between a higher BMI during childhood and an increased risk for several types of cancers in adulthood including colorectal cancer and breast cancer [106]. In comparison, there are very little data available in the GCCC region associating childhood obesity with cancer. One review reported a high prevalence of childhood overweight and obesity in Qatar, which strongly correlated with metabolic syndrome, and this was identified as a risk for adverse cardiovascular outcomes in later life [107]. There are several worldwide studies reporting that metabolic dysfunction driven by obesity including hyperglycemia and dyslipidemia is associated with an increased risk of developing at least 13 cancer types including colorectal, pancreatic, postmenopausal breast, and bladder cancers [108]. Therefore, childhood obesity in Qatar might lead to an increased rate of developing cancer later in life and must be regarded as a public health problem. Early intervention from policy makers and educators is required to raise awareness among the population to the magnitude of the obesity problem and to take necessary measures to manage this problem.

It is well established that the rate of obesity in the GCCC region is rising over the years [24,109]. A recent study found that colorectal cancer rates were increasing especially in those under the age of 50 years old. This increase has been attributed to dietary changes and a shift to a more sedentary lifestyle due to the rapid changes in the region [110]. In Kuwait, a study showed individuals were 4.3 times more likely to develop colorectal cancer if obese compared to the control group [84]. In Saudi Arabia, it was found that a high BMI was significantly associated with ovarian cancer [50]. In Qatar, a single study found that the mean BMI was 27.1 for those with gallbladder cancer, suggesting for the first time in Qatar the link between being overweight and gallbladder cancer [55].

When looking at the factors that are associated with the increase in obesity levels, it was shown that 69.2% of Qatar residents dined out or had take-away food two or more times each week in comparison to just 32.1% UK residents with total calorie intake shown to have a positive influence on cancer incidence [24,111]. Whilst diet is a well-known attributing factor of obesity, the statistics suggest that stress is another factor in the UK. It was reported that 79% of UK adults had stress periods at least once a month, and stress has been shown to affect behaviors such as inducing overeating and the consumption of foods high in fat and sugar [112,113]. There are also links to poor sleep, changes in energy homeostasis, and obesity [114]. A recent study highlighted that 68.9% of students that had moved from the gulf region to the UK to study had perceived a significant change in their diet with the consumption of sugary drinks, sweets, chocolates, cakes, and doughnuts increasing brought about by food availability and time constraints [115]. The West has undeniably influenced the GCCCs’ lifestyle in a variety of ways. Since oil was first discovered in the region with countries such as the United States, the UK, and European nations playing a crucial role in developing the GCCCs’ oil industry, many expatriate workers have called the GCCC home. Western movies and entertainment centers and social trends have all had an impact on the modern Gulf lifestyle, causing the region to become more cosmopolitan. This fusion of Western and local cultures has resulted in a unique lifestyle. Western fast-food chains are a common sight in most GCCCs, reflecting the influence of the West on dining habits. These fast-food chains are not only popular among expatriates but also among locals, leading to a fusion of Western food mixed with Arabic flavors such as Za’atar. This adoption of a more Western-style diet has been shown to reduce micronutrient levels, which have protective properties, and replace it with processed food, red meat, and trans fats leading to an increase in cancers such as colorectal cancer [116]. It is possible if this continued fast-paced adoption of Western-style diets persists, it could have a negative impact on cancer incidence in the GCCCs.

In the present study, the link between physical activity as a single factor and absence of cancer was insignificant. This contrasts with other studies that demonstrated the relationship between physical activity and the reduced incidence of certain cancers such as bladder, colon, breast, endometrial, esophageal, and gastric cardia [18,117]. The discrepancy could be attributed to a small number of studies included in this meta-analysis. Additionally, it is unclear in some studies how the participants were selected and unclear what frequency, duration, and intensity of exercise are required for primary cancer prevention or for lowering the risk/incidence of cancer. For example, a study by Al-Mhanna et al. (2024) reported that a combined aerobic and resistance training improved cancer-related indicators in breast cancer patients and survivors with obesity and/or overweight [118]. A study conducted on participants of the UK Biobank found there to be a higher mortality rate (4.4 per 1000) for those who did no vigorous physical activity in comparison to those who did more than 60 min of vigorous activity a week (0.3 per 1000) [62]. Our previous study showed that 30.1% of Qatar’s residence who were obese did no exercise at all in comparison to 7.1% of UK residence [24]. This and other dietary habits could potentially contribute to the high levels of colon cancer in the GCCC region observed in the present study. More often, it is seen that the results of leading a sedentary lifestyle are grouped into studies looking at the effects of general unhealthy lifestyles alongside diet, stress, and unbalanced sleep patterns, with between 30 and 40% of cancers being preventable through modifications to these lifestyle factors [18,119].

Exercise and physical activity have been linked to a reduction in the risk of 11 types of cancers through mechanisms such as the inhibition of tumor growth, reducing oxidative stress, increasing telomere length and improved endogenous sex hormone regulation as well as improving cancer treatment efficacy [18,120,121,122]. In bladder cancer, it was shown to enhance immune function, reduce chronic inflammation, and enhance DNA repair [57]. In breast, endometrial, ovarian, and lung cancers, physical activity may modulate the production, metabolism, and excretion of endogenous sex hormones [13,117,123,124]. Insulin, prostaglandin, and bile acid levels, which influence the growth and proliferation of colonic cells, were shown to be decreased in those undertaking physical activity [125]. Observational studies found that athletes had lowering circulating levels of testosterone, lowering the risk of prostate cancer [126]. A reduction in abdominal fat showed a metabolic improvement in glucose tolerance and insulin sensitivity in pancreatic cancer risk [127]. Physical activity may reduce blood pressure, insulin resistance, and lipid peroxidation, leading to a reduced risk of renal cancer [128]. It was also shown that physical activity reduces fasting insulin C-peptide and IGF-1, as well as lowering inflammatory cytokines, and increases adiponectin, reducing the risk of gastric and esophageal cancers [129,130].

Taken together, our meta-analysis highlights the shortage of cancer epidemiology studies from the GCCC region in comparison to studies available from the UK. The association between obesity and cancer incidence in the GCCCs is lower than in the UK, and so is the age-standardized cancer rate. Our study draws attention to the importance of comparing lifestyle factors associated with cancer in two regions that differ dramatically in their cancer incidence. The GCCC region is recommended to avoid adopting a stressful fast-life pace that promotes frequent use of fast food, characteristic to the Western region, and which leads to obesity. This study demonstrates the need for underrepresented countries to adopt their own specific health policy and can be used as a model going forward for other regions, such as South America. Future research should be directed towards studying comprehensively the unique region-specific genetic and lifestyle characteristics, dietary habits, and cultural practices that may contribute to cancer incidence.

### Limitations

Our study has several limitations: First, heterogeneity was a major factor in most of the studies, which could explain the variation in the outcomes observed. The sources of heterogeneity observed within the groups were age, cancer type, and sex, as well as the fewer number of studies from the GCCCs. We have used robust statistical tests to assess the heterogeneity. Secondly, despite the number of publications from the GCCC region increasing in the past 25 years, the scientific output is still relatively low in comparison to other countries with a smaller population [76]. Thirdly, we only included studies published in English, which makes it possible that some studies were published in Arabic in local journals that may have been omitted. Fourth, the use of BMI, although universal, is not accurate as there are variations for different ethnic groups, which could place participants into the bracket of obesity incorrectly.

The fifth limitation is disease state. It was not mentioned in the studies whether participants suffered from other illness or disease (other than cancer) that may impact their ability to engage in physical activity. All these can be impacting factors to the effect of physical activity on cancer, which were not always reported in the studies included in our meta-analysis.

## 5. Conclusions

This study showed that obesity was associated with cancer incidence in both the UK and the GCCCs, with a more significant link to cancer in the UK. Whereas there was no significant relationship between physical activity, as a single factor, and cancer incidence in either region, the presence of both obesity and lack of physical activity was significantly associated with cancer incidence. Our study has identified gaps in the research from the GCCCs, highlighting the need for further studies such as longitudinal studies. Region-specific data can be used to guide evidence-based policies aimed at preventing obesity and increasing physical activity to reduce cancer risk. This meta-analysis highlights the need to examine the modifiable lifestyle factors to address the global rise in obesity and its association with cancer so that populations residing in less-studied regions (GCCCs) can benefit from research conducted in well-characterized regions to reduce life-style-associated cancer disparities.

## Figures and Tables

**Table 2:** Summary of the data from the GCCCs and UK compared for this study.

Region	Hazard Ratio	95% CI	*p* Value
All Regions (GCCCs and UK)	0.12	0.06–0.18	<0.0001(r)
UK	0.14	0.03–0.18	<0.0001(c)
GCCCs	0.15	0.05–0.25	0.0042(r)
GCCCs Excluding Saudi Arabia	0.19	0.06–0.31	0.0030(r)
Saudi Arabia	0.37	0.36–0.38	<0.0001(c)

CI: confidence interval, (r) = random-effects model, (c) = common-effects model.

## Data Availability

All data included in this meta-analysis are provided in the article and as Supplementary Files.

## Supplementary Materials

The following supporting information can be downloaded at https://www.mdpi.com/article/10.3390/cancers16244205/s1. Supplementary Figure S1: Risk of Bias (RoB) [40,41,42,43,44,45,46,47,48,49,50,51,52,53,54,55,56,57,58,59,60,61,62,63,64,65,66,67,68,69,70,71,72,73,74,75,76,77,78,79,80,81,82,83,84,85,86,87,88,89,90,91,92,93,94,95,96,97,98,99,100,101,102]. Supplementary Figure S2: The association of age groups and cancer incidence; 30–50 [40,41,44,46,50,55,58,62,63,72,75,76,80,93,95,98,102], 50–70 [48,53,66,73,82,100], unspecified age [42,45,47,49,52,54,56,57,59,60,61,67,69,71,77,78,79,85,86,89,92,94,97] and wide range [42,45,47,49,52,54,56,57,59,60,61,67,69,71,77,78,79,85,86,89,92,94,97]. Supplementary Figure S3: The association between different types of cancers and obesity; endocrine [41,45,48,52,60,72], hematologic [60,72,78], nonspecific [42,58,62,65,71], respiratory [40,41,45,61,69,72,75,76,78,79], neurological [45,48,60,72], genitouria [40,41,45,46,48,50,57,59,60,64,68,72,74,76,78], head and neck [41,45,48,61,72], skin and soft tissue [45,48,61,72]. Supplementary Table S1a: Prisma checklist [36], Supplementary Table S1b: Prisma abstract checklist [36]. Supplementary Table S2: Components of PICO framework. Supplementary Table S3: Databases search strategy. Supplementary Table S4: GRADE table.
