# Supplementary material for: Obesity, Physical Activity, and Cancer Incidence in Two Geographically Distinct Populations; The Gulf Cooperation Council Countries and the United Kingdom—A Systematic Review and Meta-Analysis
Source: Cancers (Basel). 2024 Dec 17;16(24):4205. doi: 10.3390/cancers16244205 (PMC11674634; doi:10.3390/cancers16244205)
Supplement: Supplementary file 1 [file cancers-16-04205-s001.zip › cancers-3270190-supplementary/Suppl. Figure 1.pdf]

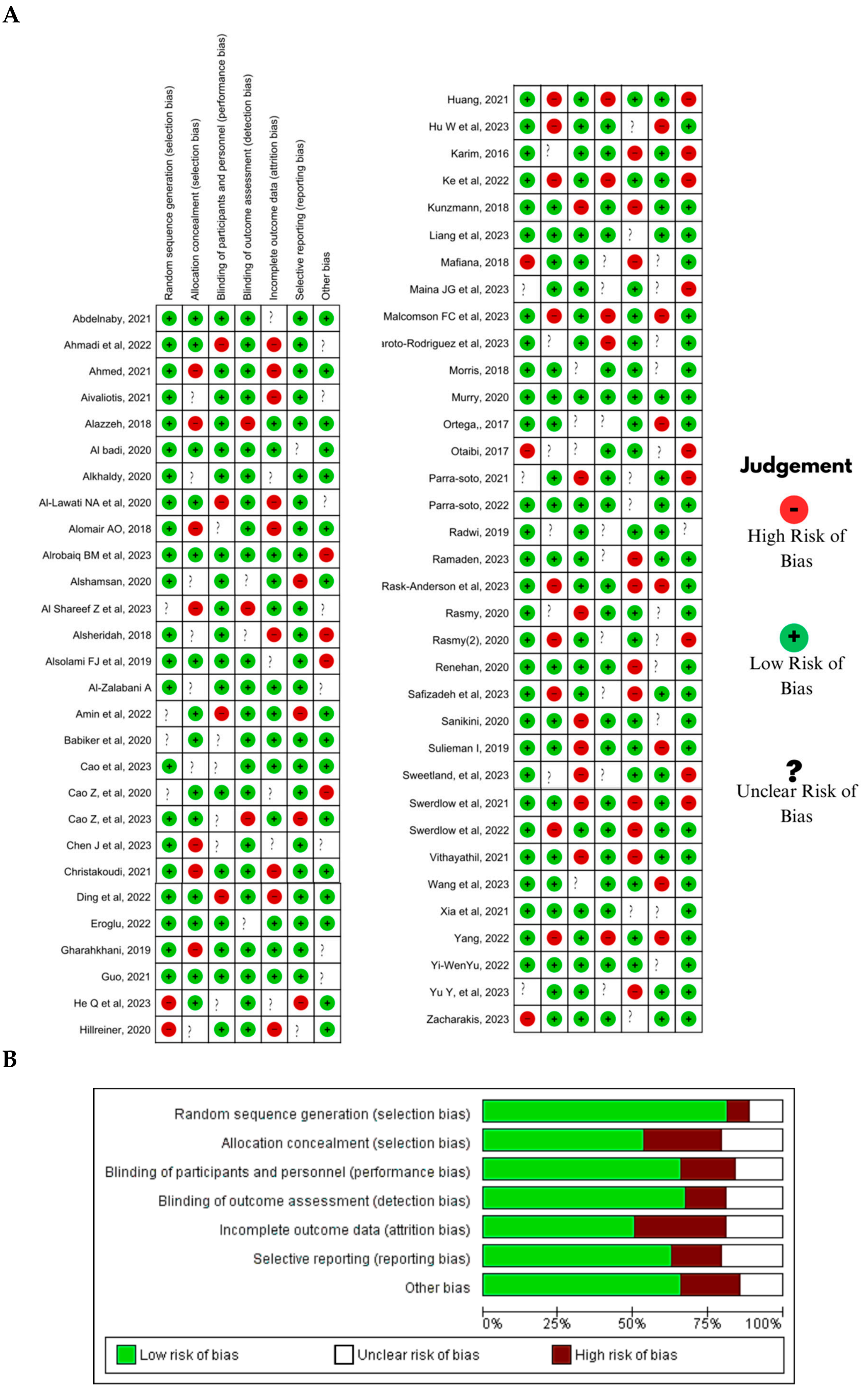
A
Random sequence generation (selection bias)
Allocation concealment (selection bias)
Blinding of participants and personnel (performance bias)
Blinding of outcome assessment (detection bias)
Incomplete outcome data (attrition bias)
Selective reporting (reporting bias)
Other bias
Abdelnaby, 2021
Ahmadi et al, 2022
Ahmed, 2021
Aivaliotis, 2021
Alazzeh, 2018
Al badi, 2020
Alkhaldy, 2020
Al-Lawati NA et al, 2020
Alomair AO, 2018
Alrobaiq BM et al, 2023
Alshamsan, 2020
Al Shareef Z et al, 2023
Alsheridah, 2018
Alsolami FJ et al, 2019
Al-Zalabani A
Amin et al, 2022
Babiker et al, 2020
Cao et al, 2023
Cao Z, et al, 2020
Cao Z, et al, 2023
Chen J et al, 2023
Christakoudi, 2021
Ding et al, 2022
Eroglu, 2022
Gharahkhani, 2019
Guo, 2021
He Q et al, 2023
Hillreiner, 2020
Huang, 2021
Hu W et al, 2023
Karim, 2016
Ke et al, 2022
Kunzmann, 2018
Liang et al, 2023
Mafiana, 2018
Maina JG et al, 2023
Malcomson FC et al, 2023
aroto-Rodriguez et al, 2023
Morris, 2018
Murry, 2020
Ortega,, 2017
Otaibi, 2017
Parra-soto, 2021
Parra-soto, 2022
Radwi, 2019
Ramaden, 2023
Rask-Anderson et al, 2023
Rasmy, 2020
Rasmy(2), 2020
Renehan, 2020
Safizadeh et al, 2023
Sanikini, 2020
Sulieman I, 2019
Sweetland, et al, 2023
Swerdlow et al, 2021
Swerdlow et al, 2022
Vithayathil, 2021
Wang et al, 2023
Xia et al, 2021
Yang, 2022
Yi-WenYu, 2022
Yu Y, et al, 2023
Zacharakis, 2023
Judgement
High Risk of Bias
Low Risk of Bias
Unclear Risk of Bias
B
Random sequence generation (selection bias)
Allocation concealment (selection bias)
Blinding of participants and personnel (performance bias)
Blinding of outcome assessment (detection bias)
Incomplete outcome data (attrition bias)
Selective reporting (reporting bias)
Other bias
0%
25%
50%
75%
100%
Low risk of bias
Unclear risk of bias
High risk of bias

Supplementary Figure 1.
The Risk of Bias (RoB) summary plot (A) offers a clear visual summary of the methodological quality of all included studies. Each study is represented by a row, with columns indicating various bias domains, including selection bias, performance bias, detection bias, attrition bias, and reporting bias. The graph (B) displays the proportion of studies with low, unclear, or high risk of bias for each domain of the Cochrane Risk of Bias tool. Each bar in the graph represents a specific bias domain, with the length of the colored sections indicating the percentage of studies falling into each risk category [40–102].

Colour coding: Green represents low risk of bias, white indicates uncertain/unclear risk of bias, and red denotes high risk of bias.
