# Supplementary material for: Obesity, Physical Activity, and Cancer Incidence in Two Geographically Distinct Populations; The Gulf Cooperation Council Countries and the United Kingdom—A Systematic Review and Meta-Analysis
Source: Cancers (Basel). 2024 Dec 17;16(24):4205. doi: 10.3390/cancers16244205 (PMC11674634; doi:10.3390/cancers16244205)
Supplement: Supplementary file 1 [file cancers-16-04205-s001.zip › cancers-3270190-supplementary/Suppl. Figure 2.pdf]

Age Group 30 - 50

Study	logHR	SE(logHR)	HR	95%–CI	Weight (common)	Weight (random)
Abdelnaby, 2021	−0.8440	0.4306	0.43	[0.18; 1.00]	0.9%	18.8%
Gharahkhani, 2019	−0.5621	0.2868	0.57	[0.32; 1.00]	2.1%	29.3%
Yu Y, et al, 2023	−0.0834	0.0425	0.92	[0.85; 1.00]	96.9%	51.9%
**Common effect model**			**0.90**	**[0.83; 0.98]**	**100.0%**	.
**Random effects model (HK)**			**0.69**	**[0.27; 1.78]**	.	**100.0%**
**Prediction interval**				**[0.00; 105.42]**		

Hazard Ratio axis: 0.01 0.1 1 10 100

Heterogeneity: $I^2 = 65\%$, $\tau^2 = 0.1023$, $p = 0.06$

Age Group 50 - 70

Study	logHR	SE(logHR)	HR	95%–CI	Weight (common)	Weight (random)
Ahmadi et al, 2022	−0.1393	0.0711	0.87	[0.76; 1.00]	3.8%	9.4%
Al Shareef Z et al, 2023	0.3001	0.1531	1.35	[1.00; 1.82]	0.8%	4.4%
Alkhaldy, 2020	−0.8916	0.4549	0.41	[0.17; 1.00]	0.1%	0.7%
Alrobaiq BM et al, 2023	0.6366	0.3248	1.89	[1.00; 3.57]	0.2%	1.3%
Huang, 2021	−0.0619	0.0316	0.94	[0.88; 1.00]	19.1%	12.4%
Ke et al, 2022	−0.4463	0.2277	0.64	[0.41; 1.00]	0.4%	2.4%
Lawati, 2018	−0.1625	0.0829	0.85	[0.72; 1.00]	2.8%	8.4%
Liang et al, 2023	−0.0408	0.0208	0.96	[0.92; 1.00]	43.8%	13.0%
Malcomson FC et al, 2023	−0.0726	0.0370	0.93	[0.86; 1.00]	13.9%	12.0%
Otaibi, 2017	−0.7550	0.3852	0.47	[0.22; 1.00]	0.1%	1.0%
Parra–soto, 2021	−0.1054	0.0538	0.90	[0.81; 1.00]	6.6%	10.8%
Rasmy, 2020	−1.2730	0.6495	0.28	[0.08; 1.00]	0.0%	0.4%
Rasmy, 2020	−0.8440	0.4306	0.43	[0.18; 1.00]	0.1%	0.8%
Sulieman I, 2019	−0.2107	0.1075	0.81	[0.66; 1.00]	1.6%	6.7%
Sweetland, et al, 2023	0.1133	0.0578	1.12	[1.00; 1.25]	5.7%	10.4%
Vithayathil, 2021	−0.2877	0.1468	0.75	[0.56; 1.00]	0.9%	4.7%
Yi–WenYu, 2022.	−0.6539	0.3336	0.52	[0.27; 1.00]	0.2%	1.3%
**Common effect model**			**0.94**	**[0.92; 0.97]**	**100.0%**	.
**Random effects model (HK)**			**0.91**	**[0.81; 1.02]**	.	**100.0%**
**Prediction interval**				**[0.71; 1.16]**		

Hazard Ratio axis: 0.1 0.5 1 2 10

Heterogeneity: $I^2 = 66\%$, $\tau^2 = 0.0115$, $p < 0.01$

Age Group Unspecified

Study	logHR	SE(logHR)	HR	95%–CI	Weight (common)	Weight (random)
Aivaliotis, 2021	−0.0943	0.0481	0.91	[0.83; 1.00]	29.3%	28.4%
Al–Zalabani A	−0.2107	0.1075	0.81	[0.66; 1.00]	5.9%	18.7%
Alshamsan, 2020	−3.5066	1.7891	0.03	[0.00; 1.00]	0.0%	0.2%
Chen J et al, 2023	0.0677	0.0345	1.07	[1.00; 1.14]	57.0%	30.3%
Hillreiner, 2020	−0.9943	0.5073	0.37	[0.14; 1.00]	0.3%	1.8%
Yang, 2022	−0.1863	0.0951	0.83	[0.69; 1.00]	7.5%	20.6%
**Common effect model**			**0.98**	**[0.93; 1.03]**	**100.0%**	.
**Random effects model (HK)**			**0.90**	**[0.70; 1.15]**	.	**100.0%**
**Prediction interval**				**[0.60; 1.34]**		

Hazard Ratio axis: 0.001 0.1 1 10 1000

Heterogeneity: $I^2 = 78\%$, $\tau^2 = 0.0154$, $p < 0.01$

Age Group Wide Range

Study	logHR	SE(logHR)	HR	95%–CI	Weight (common)	Weight (random)
Ahmed, 2021	−0.3857	0.1968	0.68	[0.46; 1.00]	0.3%	3.5%
Al–Lawati NA et al, 2020	−0.2485	0.1268	0.78	[0.61; 1.00]	0.8%	5.5%
Alazzeh, 2018	−0.3425	0.1747	0.71	[0.50; 1.00]	0.4%	4.0%
Alomair AO, 2018	−0.5978	0.3050	0.55	[0.30; 1.00]	0.1%	1.9%
Alsolami FJ et al, 2019	−0.7765	0.3962	0.46	[0.21; 1.00]	0.1%	1.2%
Cao et al, 2023	−0.1985	0.1013	0.82	[0.67; 1.00]	1.2%	6.5%
Cao Z, et al, 2020	−0.1744	0.0890	0.84	[0.71; 1.00]	1.6%	7.0%
Cao Z, et al, 2023	−0.1508	0.0770	0.86	[0.74; 1.00]	2.1%	7.5%
He Q et al, 2023	−0.1508	0.0770	0.86	[0.74; 1.00]	2.1%	7.5%
Hu W et al, 2023	0.4187	0.2136	1.52	[1.00; 2.31]	0.3%	3.1%
Karim, 2016	−2.9957	1.5284	0.05	[0.00; 1.00]	0.0%	0.1%
Kunzmann, 2018	−0.7340	0.3745	0.48	[0.23; 1.00]	0.1%	1.3%
Mafiana, 2018	−0.4155	0.2120	0.66	[0.44; 1.00]	0.3%	3.2%
Maina JG et al, 2023	0.0770	0.0393	1.08	[1.00; 1.17]	8.0%	8.9%
Maroto–Rodriguez et al, 2023	−0.1863	0.0951	0.83	[0.69; 1.00]	1.4%	6.8%
Murry, 2020	−0.5447	0.2779	0.58	[0.34; 1.00]	0.2%	2.1%
Parra–soto, 2022	−0.0408	0.0208	0.96	[0.92; 1.00]	28.6%	9.3%
Ramaden, 2023	−1.0788	0.5504	0.34	[0.12; 1.00]	0.0%	0.7%
Rask–Anderson et al, 2023	−0.0305	0.0155	0.97	[0.94; 1.00]	51.4%	9.4%
Renehan, 2020	−0.5978	0.3050	0.55	[0.30; 1.00]	0.1%	1.9%
Sanikini, 2020	−0.2485	0.1268	0.78	[0.61; 1.00]	0.8%	5.5%
Swerdlow et al, 2022	−0.6349	0.3239	0.53	[0.28; 1.00]	0.1%	1.7%
Wang et al, 2023	−0.6931	0.3536	0.50	[0.25; 1.00]	0.1%	1.5%
**Common effect model**			**0.95**	**[0.93; 0.97]**	**100.0%**	.
**Random effects model (HK)**			**0.82**	**[0.73; 0.92]**	.	**100.0%**
**Prediction interval**				**[0.59; 1.14]**		

Hazard Ratio axis: 0.01 0.1 1 10 100

Heterogeneity: $I^2 = 69\%$, $\tau^2 = 0.0227$, $p < 0.01$

Supplementary Figure 2. The association of age groups and cancer incidence. For the age group 30-50, the random effect model yielded an effect size of 0.69 with a 95% confidence interval (CI) of 0.27 to 1.78. The heterogeneity among studies had an I^2 statistic of 65% (p 0.06). The common-effects model provided an effect size of 0.90 (95% CI: 0.27 to 1.78). Most individual study estimates are clustered near the line of no effect (indicated at 1), emphasizing the consistency of findings across studies [40,41,44,46,50,55,58,62,63,72,75,76,80,93,95,98,102]. The age group 50-70 had a random effect size of 0.91 with a 95% confidence interval of 0.81 to 1.02. The heterogeneity among studies was moderate, with an I2 statistic of 66% (p < 0.01), suggesting that approximately 66% of the variability in effect estimates is due to heterogeneity rather than chance. The common-effects model provided an effect size of 0.94 (95% CI: 0.92 to 0.97) [48,53,66,73,82,100]. Age group unspecified random effect model yielded an effect size of 0.90 with a 95% confidence interval (CI) of 0.70 to 1.15. The heterogeneity among studies was high, with an I2 statistic of 78% (p < 0.01. The common-effects model provided an effect size of 0.98 (95% CI: 0.93 to 1.03) [42,45,47,49,52,54,56,57,59–61,67,69,71,77–79,85,86,89,92,94,97]. For the age group wide range, the random effect model produced an effect size of 0.82 with a 95% confidence interval (CI) of 0.73 to 0.92, indicating a statistically significant reduction in cancer incidence for this age grouping. The heterogeneity among studies was moderate, with an I2 statistic of 69% (p < 0.01), suggesting that approximately 69% of the variability in effect estimates is due to heterogeneity rather than chance. The common-effects model provided an effect size of 0.95 (95% CI: 0.93 to 0.97), The diamond at the bottom of the plots represents the overall pooled effect size, with its width reflecting the 95% CI 42,45,47,49,52,54,56-57,59-61,67,69,71,77-79,85-86,89,92,94,97].
