# Supplementary material for: Obesity, Physical Activity, and Cancer Incidence in Two Geographically Distinct Populations; The Gulf Cooperation Council Countries and the United Kingdom—A Systematic Review and Meta-Analysis
Source: Cancers (Basel). 2024 Dec 17;16(24):4205. doi: 10.3390/cancers16244205 (PMC11674634; doi:10.3390/cancers16244205)
Supplement: Supplementary file 1 [file cancers-16-04205-s001.zip › cancers-3270190-supplementary/Suppl. Figure 3.pdf]

Cancer - Endocrine

Study	logHR	SE(logHR)	HR	95%-CI	Weight (common)	Weight (random)
Al-Lawati NA et al, 2020	−0.2485	0.1268	0.78	[0.61; 1.00]	1.1%	9.0%
Cao Z, et al, 2023	−0.1508	0.0770	0.86	[0.74; 1.00]	2.9%	15.2%
Chen J et al, 2023	0.0677	0.0345	1.07	[1.00; 1.14]	14.4%	22.5%
Rask-Anderson et al, 2023	−0.0305	0.0155	0.97	[0.94; 1.00]	71.3%	24.9%
Vithayathil, 2021	−0.2877	0.1468	0.75	[0.56; 1.00]	0.8%	7.3%
Yu Y, et al, 2023	−0.0834	0.0425	0.92	[0.85; 1.00]	9.5%	21.2%
**Common effect model**			**0.97**	**[0.95; 1.00]**	**100.0%**	**.**
**Random effects model (HK)**			**0.93**	**[0.82; 1.05]**	**.**	**100.0%**
**Prediction interval**				**[0.69; 1.24]**		

Hazard Ratio axis: 0.75, 1, 1.5

Heterogeneity: $I^2 = 72\%$, $\tau^2 = 0.0087$, $p < 0.01$

Cancer - Hematologic

Study	logHR	SE(logHR)	HR	95%-CI	Weight (common)	Weight (random)
Cao Z, et al, 2020	−0.1744	0.0890	0.84	[0.71; 1.00]	37.0%	37.0%
Cao Z, et al, 2023	−0.1508	0.0770	0.86	[0.74; 1.00]	49.4%	49.4%
Vithayathil, 2021	−0.2877	0.1468	0.75	[0.56; 1.00]	13.6%	13.6%
**Common effect model**			**0.84**	**[0.75; 0.93]**	**100.0%**	**.**
**Random effects model (HK)**			**0.84**	**[0.73; 0.96]**	**.**	**100.0%**
**Prediction interval**				**[0.42; 1.66]**		

Hazard Ratio axis: 0.5, 1, 2

Heterogeneity: $I^2 = 0\%$, $\tau^2 = 0$, $p = 0.71$

Cancer - Nonspecific

Study	logHR	SE(logHR)	HR	95%-CI	Weight (common)	Weight (random)
Ahmadi et al, 2022	−0.1393	0.0711	0.87	[0.76; 1.00]	7.5%	27.1%
Ahmed, 2021	−0.3857	0.1968	0.68	[0.46; 1.00]	1.0%	7.3%
Ding et al, 2022	−0.8210	0.4189	0.44	[0.19; 1.00]	0.2%	1.8%
Liang et al, 2023	−0.0408	0.0208	0.96	[0.92; 1.00]	87.1%	43.3%
Maroto-Rodriguez et al, 2023	−0.1863	0.0951	0.83	[0.69; 1.00]	4.2%	20.5%
**Common effect model**			**0.94**	**[0.91; 0.98]**	**100.0%**	**.**
**Random effects model (HK)**			**0.87**	**[0.73; 1.05]**	**.**	**100.0%**
**Prediction interval**				**[0.63; 1.21]**		

Hazard Ratio axis: 0.2, 0.5, 1, 2, 5

Heterogeneity: $I^2 = 60\%$, $\tau^2 = 0.0073$, $p = 0.04$

Cancer - Respiratory

Study	logHR	SE(logHR)	HR	95%-CI	Weight (common)	Weight (random)
Cao Z, et al, 2020	−0.1744	0.0890	0.84	[0.71; 1.00]	1.4%	1.9%
Cao Z, et al, 2023	−0.1508	0.0770	0.86	[0.74; 1.00]	1.9%	2.6%
Huang, 2021	−0.0619	0.0316	0.94	[0.88; 1.00]	11.0%	13.4%
Malcomson FC et al, 2023	−0.0726	0.0370	0.93	[0.86; 1.00]	8.0%	10.2%
Murry, 2020	−0.5447	0.2779	0.58	[0.34; 1.00]	0.1%	0.2%
Parra-soto, 2022	−0.0408	0.0208	0.96	[0.92; 1.00]	25.4%	25.7%
Rask-Anderson et al, 2023	−0.0305	0.0155	0.97	[0.94; 1.00]	45.6%	37.4%
Vithayathil, 2021	−0.2877	0.1468	0.75	[0.56; 1.00]	0.5%	0.7%
Yu Y, et al, 2023	−0.0834	0.0425	0.92	[0.85; 1.00]	6.1%	7.9%
**Common effect model**			**0.95**	**[0.93; 0.97]**	**100.0%**	**.**
**Random effects model (HK)**			**0.95**	**[0.91; 0.98]**	**.**	**100.0%**
**Prediction interval**				**[0.91; 0.99]**		

Hazard Ratio axis: 0.5, 1, 2

Heterogeneity: $I^2 = 35\%$, $\tau^2 = 0.0002$, $p = 0.14$

Cancer - Neurologic

Study	logHR	SE(logHR)	HR	95%-CI	Weight (common)	Weight (random)
Cao Z, et al, 2023	−0.1508	0.0770	0.86	[0.74; 1.00]	3.3%	22.8%
Chen J et al, 2023	0.0677	0.0345	1.07	[1.00; 1.14]	16.2%	31.3%
Rask-Anderson et al, 2023	−0.0305	0.0155	0.97	[0.94; 1.00]	79.7%	33.9%
Vithayathil, 2021	−0.2877	0.1468	0.75	[0.56; 1.00]	0.9%	12.0%
**Common effect model**			**0.98**	**[0.95; 1.01]**	**100.0%**	**.**
**Random effects model (HK)**			**0.94**	**[0.76; 1.17]**	**.**	**100.0%**
**Prediction interval**				**[0.55; 1.61]**		

Hazard Ratio axis: 0.75, 1, 1.5

Heterogeneity: $I^2 = 77\%$, $\tau^2 = 0.0114$, $p < 0.01$

Cancer - Genitourina

Study	logHR	SE(logHR)	HR	95%-CI	Weight (common)	Weight (random)
Al Shareef Z et al, 2023	0.3001	0.1531	1.35	[1.00; 1.82]	0.6%	3.0%
Alrobaiq BM et al, 2023	0.6366	0.3248	1.89	[1.00; 3.57]	0.1%	0.8%
Amin et al, 2022	−0.1744	0.0890	0.84	[0.71; 1.00]	1.7%	6.4%
Cao Z, et al, 2020	−0.1744	0.0890	0.84	[0.71; 1.00]	1.7%	6.4%
Cao Z, et al, 2023	−0.1508	0.0770	0.86	[0.74; 1.00]	2.3%	7.4%
Chen J et al, 2023	0.0677	0.0345	1.07	[1.00; 1.14]	11.4%	12.2%
Christakoudi, 2021	−0.2231	0.1138	0.80	[0.64; 1.00]	1.0%	4.7%
He Q et al, 2023	−0.1508	0.0770	0.86	[0.74; 1.00]	2.3%	7.4%
Malcomson FC et al, 2023	−0.0726	0.0370	0.93	[0.86; 1.00]	9.9%	11.9%
Parra-soto, 2021	−0.1054	0.0538	0.90	[0.81; 1.00]	4.7%	9.9%
Rask-Anderson et al, 2023	−0.0305	0.0155	0.97	[0.94; 1.00]	56.0%	14.0%
Vithayathil, 2021	−0.2877	0.1468	0.75	[0.56; 1.00]	0.6%	3.2%
Wang et al, 2023	−0.6931	0.3536	0.50	[0.25; 1.00]	0.1%	0.7%
Yi-WenYu, 2022.	−0.6539	0.3336	0.52	[0.27; 1.00]	0.1%	0.8%
Yu Y, et al, 2023	−0.0834	0.0425	0.92	[0.85; 1.00]	7.5%	11.2%
**Common effect model**			**0.96**	**[0.94; 0.98]**	**100.0%**	**.**
**Random effects model (HK)**			**0.92**	**[0.85; 1.00]**	**.**	**100.0%**
**Prediction interval**				**[0.77; 1.10]**		

Hazard Ratio axis: 0.5, 1, 2

Heterogeneity: $I^2 = 68\%$, $\tau^2 = 0.0062$, $p < 0.01$

Cancer - Head and Neck

Study	logHR	SE(logHR)	HR	95%-CI	Weight (common)	Weight (random)
Cao Z, et al, 2023	−0.1508	0.0770	0.86	[0.74; 1.00]	2.9%	15.8%
Chen J et al, 2023	0.0677	0.0345	1.07	[1.00; 1.14]	14.6%	25.2%
Rask-Anderson et al, 2023	−0.0305	0.0155	0.97	[0.94; 1.00]	72.6%	28.5%
Vithayathil, 2021	−0.2877	0.1468	0.75	[0.56; 1.00]	0.8%	7.1%
Yu Y, et al, 2023	−0.0834	0.0425	0.92	[0.85; 1.00]	9.6%	23.4%
**Common effect model**			**0.97**	**[0.95; 1.00]**	**100.0%**	**.**
**Random effects model (HK)**			**0.95**	**[0.83; 1.08]**	**.**	**100.0%**
**Prediction interval**				**[0.70; 1.28]**		

Hazard Ratio axis: 0.75, 1, 1.5

Heterogeneity: $I^2 = 73\%$, $\tau^2 = 0.0068$, $p < 0.01$

Cancer - Skin and Soft Tissue

Study	logHR	SE(logHR)	HR	95%-CI	Weight (common)	Weight (random)
Cao Z, et al, 2023	−0.1508	0.0770	0.86	[0.74; 1.00]	3.3%	22.8%
Chen J et al, 2023	0.0677	0.0345	1.07	[1.00; 1.14]	16.2%	31.3%
Rask-Anderson et al, 2023	−0.0305	0.0155	0.97	[0.94; 1.00]	79.7%	33.9%
Vithayathil, 2021	−0.2877	0.1468	0.75	[0.56; 1.00]	0.9%	12.0%
**Common effect model**			**0.98**	**[0.95; 1.01]**	**100.0%**	**.**
**Random effects model (HK)**			**0.94**	**[0.76; 1.17]**	**.**	**100.0%**
**Prediction interval**				**[0.55; 1.61]**		

Hazard Ratio axis: 0.75, 1, 1.5

Heterogeneity: $I^2 = 77\%$, $\tau^2 = 0.0114$, $p < 0.01$

Supplementary Figure 3. The association between different types of cancer and obesity. For the cancer subgroup Endocrine, the random effect model yielded an effect size of 0.93 with a 95% confidence interval (CI) of 0.82 to 1.05. The heterogeneity among studies was high with an I^2 statistic of 72% ($p < 0.01$). The common-effects model provided an effect size of 0.97 (95% CI: 0.95 to 1.00) [41,45,48,52,60,72]. The Hematologic subgroup had a random effect size of 0.84 with a 95% CI of 0.73 to 0.96. The heterogeneity among studies, with an I^2 statistic of 0% (p 0.71), suggesting no heterogeneity. The common-effects model provided an effect size of 0.84 (95% CI: 0.95 to 1.00) [60,72,78]. The nonspecific subgroup random effect model yielded an effect size of 0.87 with a 95% CI of 0.73 to 1.05. The heterogeneity among studies was moderate, with an I^2 statistic of 60% (p 0.04). The common-effects model provided an effect size of 0.94 (95% CI: 0.91 to 0.98) [42,58,62,65,71]. For the cancer respiratory subgroup, the random effect model produced an effect size of 0.95 with a 95% CI of 0.91 to 0.98. The heterogeneity among studies was low, with an I2 statistic of 35% (p 0.14). The common-effects model provided an effect size of 0.95 (95% CI: 0.93 to 0.97) [40,41,45,61,69,72,75,76,78,79]. The four studies for neurologic cancer had a random effect model of 0.94 with a 95% CI of 0.76 to 1.01. The heterogeneity among studies was high with an I2 statistic of 77% ($p < 0.01$), suggesting that approximately 77% of the variability in effect estimates is due to heterogeneity rather than chance [45,48,60,72]. The common-effects model provided an effect size of 0.98 (95% CI: 0.95 to 1.01). The Genitourina subgroup random effect model produced an effect size of 0.92 with a 95% CI of 0.85 to 1.00. The heterogeneity among studies was high with an I2 statistic of 68% ($p < 0.01$). The common-effects model provided an effect size of 0.96 (95% CI: 0.94 to 0.98) [40,41,45,46,48,50,57,59,60,64,68,72,74,76,78]. For the Head and Neck cancer subgroup, the random effect model showed an effect size of 0.95 with a 95% CI of 0.83 to 1.08. The heterogeneity among studies was high with an I2 statistic of 73% ($p < 0.01$). The common-effects model provided an effect size of 0.97 (95% CI: 0.95 to 1.00) [41,45,48,61,72]. The Skin and Soft Tissue subgroup random effect model yielded an effect size of 0.98 with a 95% CI of 0.95 to 1.01. The heterogeneity among studies was high with an I2 statistic of 77% ($p < 0.01$). The common-effects model provided an effect size of 0.98 (95% CI: 0.95 to 1.01). The diamond at the bottom of the plots represents the overall pooled effect size, with its width reflecting the 95% CI [45,48,61,72].
