# Supplementary material for: Obesity, Physical Activity, and Cancer Incidence in Two Geographically Distinct Populations; The Gulf Cooperation Council Countries and the United Kingdom—A Systematic Review and Meta-Analysis
Source: Cancers (Basel). 2024 Dec 17;16(24):4205. doi: 10.3390/cancers16244205 (PMC11674634; doi:10.3390/cancers16244205)
Supplement: Supplementary file 1 [file cancers-16-04205-s001.zip › cancers-3270190-supplementary/Suppl. Table 2-Components of PICO.pdf]

Supplementary Table 2: Components of the PICO framework from the current Meta-analysis

Components of PICO	
Population (P)	Individuals from two geographically distinct populations: the Gulf Cooperation Council Countries (GCCC) countries and the United Kingdom.
Intervention (I)	The presence of obesity and/or engagement in physical activity.
Comparator (C)	Comparison between cancer incidence in individuals with obesity and/or low physical activity levels versus those with normal weight and/or higher physical activity levels within the same population or between the two populations (GCCC vs. UK).
Outcomes (O)	The incidence of cancer among these populations, specifically looking at how obesity and physical activity influence cancer incidence.
Research question	What is the impact of obesity and physical activity on cancer incidence in populations from the Gulf Cooperation Council Countries (GCCC) countries compared to the United Kingdom?
