# Supplementary material for: Obesity, Physical Activity, and Cancer Incidence in Two Geographically Distinct Populations; The Gulf Cooperation Council Countries and the United Kingdom—A Systematic Review and Meta-Analysis
Source: Cancers (Basel). 2024 Dec 17;16(24):4205. doi: 10.3390/cancers16244205 (PMC11674634; doi:10.3390/cancers16244205)
Supplement: Supplementary file 1 [file cancers-16-04205-s001.zip › cancers-3270190-supplementary/Suppl. Table 3-Databases search strategy.pdf]

Database search strategies
All searches last conducted on 13 November 2023.

APA PsycINFO (Ebscohost)

Search mode: Boolean/Phrase

#	Search	Results
1	((TI bahrain* OR AB bahrain*) OR (TI kuwait* OR AB kuwait*) OR (Ti oman* OR AB oman*) OR (TI qatar* OR AB qatar*) OR (TI saudi* OR AB saudi*) OR (TI emirat* OR AB emirat*) OR (TI UAE OR AB UAE) OR (TI “gulf cooperation council" OR AB “gulf cooperation council") OR (TI "united kingdom" OR AB "united kingdom") OR (TI "channel islands" OR AB "channel islands") OR (TI guernsey OR AB guernsey) OR (TI jersey OR AB jersey) OR (TI england OR AB England) OR (TI "northern ireland" OR AB "northern ireland") OR (TI scotland OR AB scotland)) AND ((TI cancer OR AB cancer) OR (TI neoplasm* OR AB neoplasm*)) AND (((TI obes* OR AB obes*) OR (TI overweight OR AB overweight) OR (TI bmi OR AB bmi)) OR ((TI exercis* OR AB exercis*) OR (TI "aerobic activity" OR AB "aerobic activity") OR (TI "aerobic training" OR AB "aerobic training") OR (TI "anaerobic activity" OR AB "anaerobic activity") OR (TI "resistance training" OR AB "resistance training") OR (TI "strength training" OR AB "strength training") OR (TI "weight training" OR AB "weight training") OR (TI "physical activity" OR AB "physical activity") OR (TI sport* OR AB sport*) OR (TI fitness OR AB fitness) OR (TI "physical education OR AB "physical education") OR (TI "physical exertion" OR AB "physical exertion") OR (TI sedentary OR AB sedentary) OR (TI "physical inactivity" OR AB "physical inactivity")))	90

Embase

#	Search	Results
1	Bahrain/ or Kuwait/ or Oman/ or Qatar/ or Saudi Arabia/ or United Arab Emirates/ or exp United Kingdom/	513,101
2	gulf cooperation council.mp.	530
3	(bahrain* or kuwait* or oman* or qatar* or saudi arabia* or emirat* or UAE or united kingdom or channel islands or guernsey or jersey or england or northern ireland or scotland).ti,ab.	236,120
4	1 or 2 or 3	595,691
5	exp neoplasm/	5,579,940
6	(cancer* or neoplasm*).ti,ab.	3,342,519
7	5 or 6	6,192,414
8	exp obesity/	671,744
9	(obes* or overweight or bmi).ti,ab.	846,059
10	exp exercise/ or exp physical activity/ or physical inactivity/ or training/ or exp sport/ or sedentary lifestyle/ or sedentary time/ or sedentary work/	1,087,278
11	(aerobic exercis* or anaerobic exercise* or muscle strengthening exercis* or physical exercis* or resistance exercis* or aerobic training or exercise training or resistance training or strength training or weight training or aerobic activit* or anaerobic activit* or fitness or physical activit* or sport* or physical education or physical exertion or sedentary or physical inactiv*).ti,ab.	512,866
12	8 or 9 or 10 or 11	2,137,925

13	4 and 7 and 12	3,652

PubMed

#	Search	Results
1	(("gulf cooperation council"[Title/Abstract] OR "GCC"[Title/Abstract] OR "Bahrain"[MeSH Terms] OR "Kuwait"[MeSH Terms] OR "Oman"[MeSH Terms] OR "Qatar"[MeSH Terms] OR "Saudi Arabia"[MeSH Terms] OR "United Arab Emirates"[MeSH Terms] OR "bahrain*"[Title/Abstract] OR "kuwait*"[Title/Abstract] OR "oman*"[Title/Abstract] OR "qatar*"[Title/Abstract] OR "saudi arabia*"[Title/Abstract] OR "emirat*"[Title/Abstract] OR "UAE"[Title/Abstract]) OR ("United Kingdom"[Mesh] OR "United Kingdom"[tiab] OR "UK"[tiab] OR "Channel Islands"[tiab] OR "England"[tiab] OR "Guernsey"[tiab] OR "Jersey"[tiab] OR "Northern Ireland"[tiab] OR "Scotland"[tiab] OR "Wales"[tiab])) AND ("Neoplasms"[MeSH Terms] OR "cancer"[Title/Abstract] OR "cancers"[Title/Abstract] OR "neoplasm*"[Title/Abstract]) AND (("Obesity"[MeSH Terms] OR "obes*"[Title/Abstract] OR "overweight"[Title/Abstract] OR "bmi"[Title/Abstract]) OR ("Exercise"[MeSH Terms] OR "Sedentary Behavior"[MeSH Terms] OR Sports[MeSH Terms] OR "aerobic exercis*"[Title/Abstract] OR "anaerobic exercise*"[Title/Abstract] OR "muscle strengthening exercis*"[Title/Abstract] OR "physical exercis*"[Title/Abstract] OR "resistance exercis*"[Title/Abstract] OR "aerobic training"[Title/Abstract] OR "exercise training"[Title/Abstract] OR "resistance training"[Title/Abstract] OR "strength training"[Title/Abstract] OR "weight training"[Title/Abstract] OR "aerobic activit*"[Title/Abstract] OR "anaerobic activit*"[Title/Abstract] OR "physical activit*"[Title/Abstract] OR "sport*"[Title/Abstract] OR "physical education"[Title/Abstract] OR "physical exertion"[Title/Abstract] OR "fitness"[Title/Abstract] OR "sedentary"[Title/Abstract] OR "physical inactiv*"[Title/Abstract]))	1,885

Scopus

#	Search	Results
1	(TITLE-ABS-KEY (bahrain* OR kuwait* OR oman* OR qatar* OR saudi* OR emirat* OR uae OR "gulf cooperation council" OR "united kingdom" OR "channel islands" OR guernsey OR jersey OR england OR "northern ireland" OR scotland OR wales)) AND (TITLE-ABS-KEY (cancer* OR neoplasm*)) AND ((TITLE-ABS-KEY (obes* OR overweight OR bmi)) OR (TITLE-ABS-KEY (exercis* OR "aerobic activity" OR "aerobic training" OR "anaerobic activity" OR "anaerobic training" OR "resistance training" OR "strength training" OR "weight training" OR sport* OR fitness OR "physical education" OR "physical exercise" OR "physcial exertion" OR sedentary OR "physical inactivity")))	2,324
